# Identification of treatment‐induced vulnerabilities in pancreatic cancer patients using functional model systems

**DOI:** 10.15252/emmm.202114876

**Published:** 2022-02-04

**Authors:** Katja Peschke, Hannah Jakubowsky, Arlett Schäfer, Carlo Maurer, Sebastian Lange, Felix Orben, Raquel Bernad, Felix N Harder, Matthias Eiber, Rupert Öllinger, Katja Steiger, Melissa Schlitter, Wilko Weichert, Ulrich Mayr, Veit Phillip, Christoph Schlag, Roland M Schmid, Rickmer F Braren, Bo Kong, Ihsan Ekin Demir, Helmut Friess, Roland Rad, Dieter Saur, Günter Schneider, Maximilian Reichert

**Affiliations:** ^1^ Medical Clinic and Polyclinic II Klinikum rechts der Isar Technical University of Munich München Germany; ^2^ Institute for Translational Cancer Research and Experimental Cancer Therapy Technical University of Munich Munich Germany; ^3^ Institute of Molecular Oncology and Functional Genomics TUM School of Medicine Technical University of Munich Munich Germany; ^4^ Institute of Diagnostic and Interventional Radiology Technical University of Munich Munich Germany; ^5^ Department of Nuclear Medicine Klinikum Rechts der Isar Technical University of Munich Munich Germany; ^6^ Institute of Pathology Technical University of Munich München Germany; ^7^ German Cancer Research Center (DKFZ) and German Cancer Consortium (DKTK) Heidelberg Germany; ^8^ Department of Surgery Klinikum rechts der Isar Technical University of Munich Munich Germany; ^9^ Department of General Surgery University of Ulm Ulm Germany; ^10^ Department of General, Visceral and Pediatric Surgery University Medical Center Göttingen Göttingen Germany; ^11^ Center for Protein Assemblies (CPA) Technical University of Munich Garching Germany; ^12^ Translational Pancreatic Cancer Research Center Medical Clinic and Polyclinic II Klinikum rechts der Isar Technical University of Munich München Germany

**Keywords:** functional screening, pancreatic cancer, precision oncology, therapy‐induced vulnerabilities, tumor cell plasticity, Cancer, Digestive System

## Abstract

Despite the advance and success of precision oncology in gastrointestinal cancers, the frequency of molecular‐informed therapy decisions in pancreatic ductal adenocarcinoma (PDAC) is currently neglectable. We present a longitudinal precision oncology platform based on functional model systems, including patient‐derived organoids, to identify chemotherapy‐induced vulnerabilities. We demonstrate that treatment‐induced tumor cell plasticity *in vivo* distinctly changes responsiveness to targeted therapies, without the presence of a selectable genetic marker, indicating that tumor cell plasticity can be functionalized. By adding a mechanistic layer to precision oncology, adaptive processes of tumors under therapy can be exploited, particularly in highly plastic tumors, such as pancreatic cancer.

## Graphical abstract

Therapy resistance remains a major challenge in pancreatic cancer. This study used patient‐derived organoids (PDOs) and cell lines (PDCL) to investigate response to standard of care chemotherapy administered in humans. We show that chemotherapy‐induced adaption exposes novel therapeutic vulnerabilities accompanied by cellular re‐differentiation.

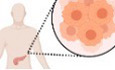

**The paper explained**

**Problem**

Pancreatic ductal adenocarcinoma (PDAC) has one of the lowest 5‐year survival rate of all cancers and is considered a highly plastic tumor. Cellular plasticity is the ability of tumor cells to adapt to changing conditions by acquiring different molecular and phenotypic identities and, thereby, plasticity programs are key regulators of acquired treatment resistance.

**Results**

By using pancreatic cancer patient‐derived organoids (PDOs) and cell lines (PDCLs) before and after standard‐of‐care polychemotherapy, we were able to demonstrate that pancreatic cancer cells show plastic behaviors under treatment‐imposed pressure in a clinical setting. Although, treatment‐naïve and ‐exposed PDO cluster within the same PDAC subtype and share genetic drivers, PDOs differ from each other phenotypically and display a certain degree epithelial reprogramming. In large‐scale drug screens, we discovered that plasticity re‐programming induces specific therapeutic vulnerabilities. Specifically, we identified a markedly increased sensitivity toward MEK inhibition in this context.

**Impact**

Here, we provide a functional precision oncology platform to identify treatment‐induced vulnerabilities in a clinically relevant timeframe. Although genomics‐informed clinical decision making has transformed clinical oncology; only a subset of PDAC patients harbors tractable alterations. Functional precision oncology will potentially increase therapeutic opportunities in the framework of personalized treatment decisions.

## Introduction

Pancreatic ductal adenocarcinoma (PDAC) will be the second cancer‐related death reason in 2040 (Rahib *et al*, 2021), and current therapies rely on conventional polychemotherapies with poor outcomes. In the minority of patients, molecular‐informed targeted therapy opportunities exist. For example, in 0.5–1% of patients with DNA mismatch‐repair lesions, immune checkpoint inhibitors are approved (Nevala‐Plagemann *et al*, 2020). Furthermore, patients with genetic fusions, including the tropomyosin receptor kinase (TRK) gene family *NTRK1,* can be treated with larotrectinib/Entrectinib (Nevala‐Plagemann *et al*, 2020). Although the value of molecular marker–selected therapies is documented in PDAC (Pishvaian *et al*, 2020), even after a complete molecular workup, three out of four patients revealed no actionable target (Pishvaian *et al*, 2020). This type of studies clearly demonstrates that the tremendous potential of genetically guided precision oncology, however, also underscores its apparent limitations.

In the last ten years, a continuously increased usage of primary cellular patient‐derived cancer models was evident. Two‐dimensional (2D) cultured PDAC models preserve relevant mutations, which remained stable up to 40 passages (Knudsen *et al*, 2018). These 2D lines are an important platform to investigate drug responses (Knudsen *et al*, 2018; Dreyer *et al*, 2021). In addition, 3D PDAC organoid models were applied to understand and to analyze the biology and therapeutic vulnerabilities of PDAC (Tiriac *et al*, 2018; Driehuis *et al*, 2019; Dantes *et al*, 2020; Iovanna, 2021). Evidence exists that PDAC organoids are clinical predictive biomarkers (Tiriac *et al*, 2018; Driehuis *et al*, 2019; Wensink *et al*, 2021). Importantly, primary PDAC models allow to implement a mechanistic layer into precision oncology by using them in genetic or pharmacological screening experiments. We report here on a longitudinal precision oncology platform, which points to the value of mechanistic investigation to advance concepts for PDAC targeting.

## Results and Discussion

**Figure EV1:**
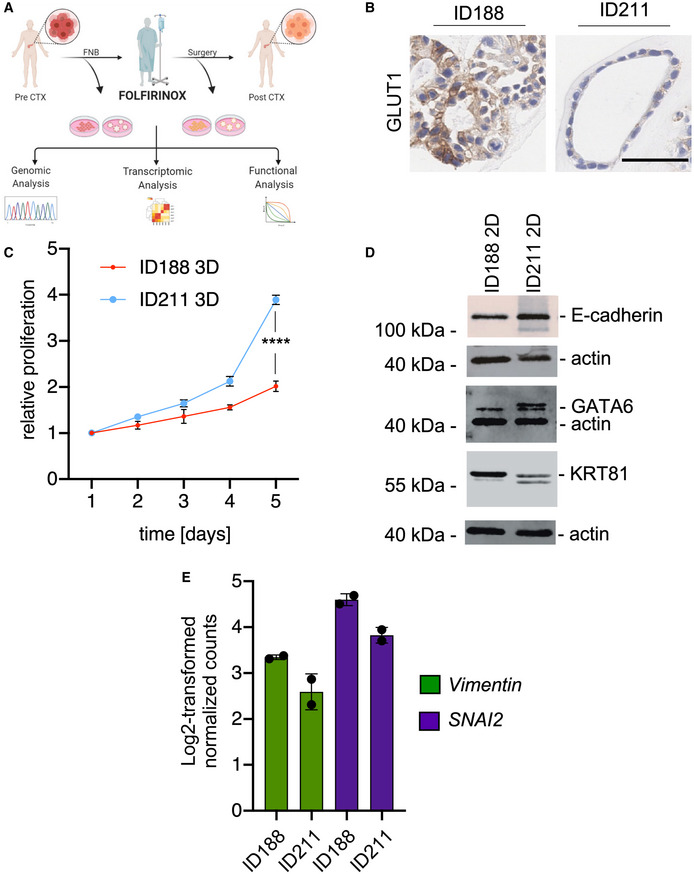
Characterization of pre‐ and post‐FFX (FOLFIRINOX) models Schematic illustration of the biopsy strategy and downstream applications.GLUT1 IHC of embedded and sectioned organoids (p30 in both PDO lines). Scale bar = 50 µm.Growth curves of ID188 (p27‐29) and ID211 (p26–28) in 3D culture measured using CellTiter Glo assay for five consecutive days. Shown is the mean ± SD of three independent replicates. Unpaired *t*‐test ****P < 0.0001.Western blots of E‐cadherin, GATA6, and KRT81 in ID188 (p5+33) and ID211 (p9+15) 2D lines. Actin = loading control. *n* = 2.
*Vimentin* and *SNAI2* mRNA expression retrieved from RNA‐seq experiments in ID188 and ID211 organoids. Shown is the mean ± SD of two technical replicates.

**Figure 1:**
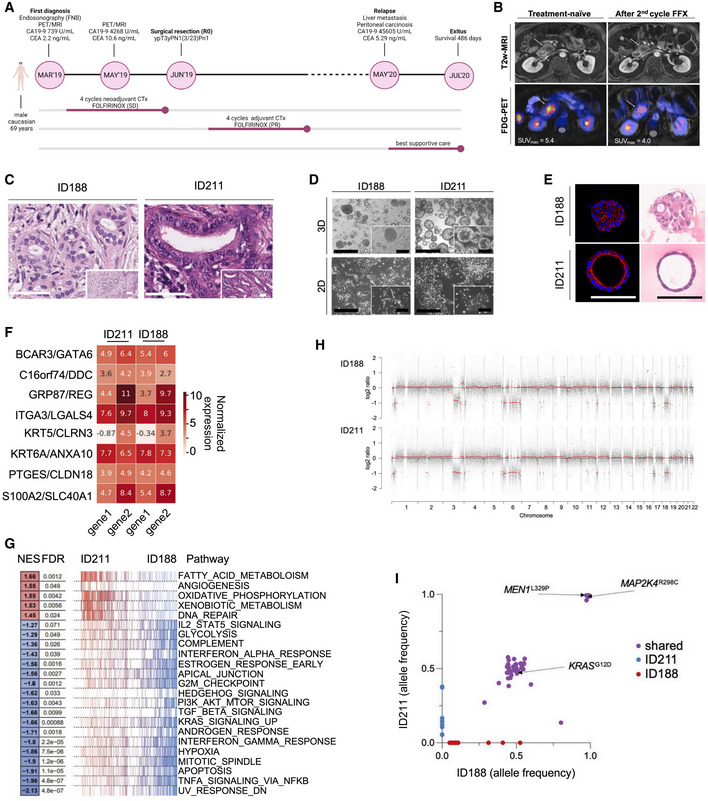
Generation and characterization of chemotherapy‐naïve and ‐exposed patient‐derived models to investigate treatment‐imposed reprogramming Clinical history of a PDAC patient receiving perioperative FOLFIRINOX including follow‐up.
^18^F‐FDG PET‐MRI at the time of the diagnosis and in the interim staging after one cycle of FOLFIRINOX.H&E staining of FNB and the resection specimen. Scale bars represent 50 µm.Phase‐contrast images of organoids (upper panel) and respective 2D cultures (isolated from the biopsy before FOLFIRINOX (ID188) or after neoadjuvant therapy from the resected cancer (ID211)). Scale bar represents 200 µm.IF staining of the actin filaments (red) and nuclei (blue) of organoids in the left panel, and H&E staining of embedded and sectioned organoids in the right panel (ID188 p30, ID211 p30). Scale bars represent 50 µm.Normalized expression (log_2_ scale) for gene pairs evaluated during purity‐independent subtyping of tumors (PurIST). Normalized expression (log_2_ scale), gene 1 = basal‐like, gene 2 = classical.Gene Set Enrichment Analysis (GSEA) of pre‐(ID188, p7) and post‐treatment (ID211, p6) PDO lines. Normalized enrichment score (NES) and the adjusted *P*‐value are depicted.Whole‐exome sequencing–based copy number profiles for ID188 (p7) and ID211 (p6) organoids.Allele frequencies of miss‐ and nonsense SNVs and Indels which are shared between both organoid lines (purple), private to ID188 (red), or private to ID211 (blue). Source data are available online for this figure.

In the framework of the German collaborative research center 1321 “Modeling and targeting pancreatic cancer”, we have established a translational oncology workflow to multidimensionally characterize PDAC biopsies, including genomic, transcriptomic, and functional analysis (Fig EV1A). This platform harbors a unique opportunity to understand adaptive processes in tumor evolution and/or treatment‐imposed pressure in PDAC patients longitudinally. Here, a male patient with borderline resectable PDAC entered the platform in March 2019 (Fig 1A). The patient was treated with four cycles neoadjuvant FOLFIRINOX. In an interim staging by ^18^F‐FDG PET‐MRI, the glucose metabolism (as measured by the standard uptake value) was markedly reduced while the tumor size was unaltered, indicating a metabolic switch (Fig 1B). The tumor was resected in June 2019 (ypT3, ypN1(3/23), Pn1, R0), and the patient continued perioperative‐modified FOLFIRINOX treatment for another four cycles. A relapse with metastasis was observed in May 2020, and the patient succumbed to the disease in July 2020 (Fig 1A). Before receiving neoadjuvant treatment, an endoscopic ultrasound‐guided fine needle biopsy (EUS‐FNB) was performed for diagnostic purposes (Fig 1C). An additional biopsy was used to generate a patient‐derived organoid (PDO) and a 2D cell line (ID188 = treatment‐naive) (Fig 1D). Another set of PDO/ 2D lines (ID211 = post‐treatment) was generated from the surgical resection specimen after FOLFIRINOX induction (Fig 1C and D). Histologically, both biopsies demonstrated a well‐to‐moderate differentiation (Fig 1C). PDO‐isolated pre‐chemotherapy (ID188) showed a lumen filling growth pattern in 3D and revealed a quasi‐mesenchymal growth in 2D (Fig 1D and E). PDOs isolated from the resection (ID211) displayed a cystic growth pattern in 3D and grew as an epithelial monolayer with colony forming growth in 2D (Fig 1D and E). A lumen‐filling phenotype was recently shown to be characteristic for human pluripotent stem cell–derived *KRAS^mut^*‐driven organoids undergoing de‐differentiation (Breunig *et al*, 2021) as well as in a subset of PDAC PDOs (Driehuis *et al*, 2019). Recent transcriptomic subtyping efforts group PDACs in more aggressive and therapy‐resistant basal‐like or classical subtypes (Collisson *et al*, 2019). We performed RNA‐seq on PDO ID188 and ID211 and applied purity‐independent subtyping of tumor (PurIST) score (Rashid *et al*, 2020). Both PDOs were classified as classical PDACs with probability of being basal‐like of less than 0.1 (ID188: 0.02073, ID211: 0.00829) as deduced from the ratios of eight classifier gene pairs (gene 1 = basal‐like, gene 2 = classical) (Fig 1F). Gene set enrichment analysis (GSEA) demonstrated distinct changes of pathways altered by chemotherapy (Fig 1G). Consistent with the reduced signal in ^18^F‐FDG PET (Fig 1B), a glycolysis signature was depleted in ID211, and oxidative phosphorylation and lipid metabolism were activated to serve the metabolic demands (Fig 1G). Consistently, expression of the glucose transporter GLUT1 was reduced in ID211 PDOs (Fig EV1B). Importantly, pathways associated with the basal‐like subtype, such as glycolysis, TGF beta signaling, cell cycle, hypoxia, or inflammation were enriched in PDO ID188 isolated before FOLFIRINOX therapy (Fig 1G). These changes in the activity of oncogenic networks, together with the growth pattern of the cellular models in which the posttreatment ID211 showed less lumen‐filling in 3D as well as epithelial colonies in 2D (Fig 1E), indicated a certain degree of re‐differentiation. This growth pattern was associated with a proliferative advantage for PDO ID211 (Fig EV1C). Evidence for re‐differentiation was additionally observed in 2D models of ID188 and ID211 by investigating protein expression of KRT81, E‐cadherin, and GATA6 (Fig EV1D). Furthermore, in the PDOs, mRNA expression of *Vim* and the EMT transcription factor *SNAI2* was reduced in ID211 (Fig EV1E). To determine whether these therapy‐induced changes could be explained genetically or on a level of cellular plasticity, we analyzed copy number variations (CNVs) and single‐nucleotide variants (SNVs) in PDOs ID188 and ID211. Surprisingly, over all chromosomes, genetic gains and losses were similar (Fig 1H), with the tumor driven by a *KRAS^G12D^* mutation and additional mutations in *MEN1^L329P^* and *MAP2K4* *^R298C^*, both rare genetic events in PDAC. No mutations in *TP53* or *SMAD4* were observed (Fig 1I and Dataset EV1 and EV2). Importantly, SNVs with a relevant mutant allele frequency are highly concordant in the investigated models (Fig 1I and Dataset EV1 and EV2). In summary, despite a clear change of the biology of the tumor upon chemotherapy, as evidenced by reduced glucose uptake *in vivo* with consistent changes in metabolic signatures *ex vivo*, the genetic landscape of the models is not changed, indicating that tumor cell plasticity drives these adaptive processes.

**Figure 2:**
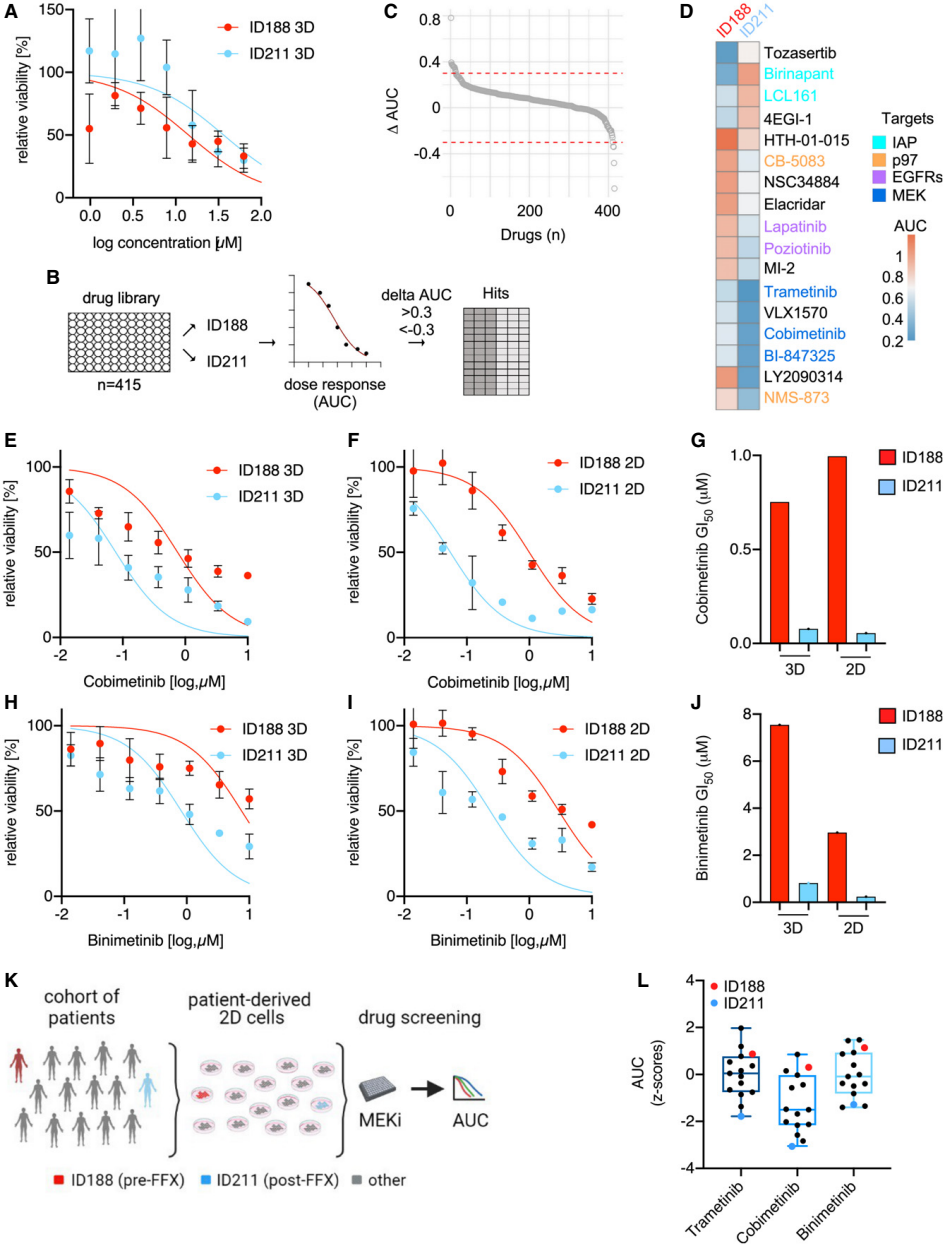
Large‐scale drug screens reveal selective FOLFIRINOX‐induced vulnerabilities ADose– response curves of ID188 (p27–29) and ID211 (p26–28) organoids treated with FOLFIRINOX over three days. ATP was measured with CellTiter‐Glo assays. Shown is the mean ± SD of three independent experiments.BSchematic illustration of the unbiased drug screening experiment.CPlot of the delta AUC in ID188 (p5+27) versus ID211 (p9+11) cells over 415 drugs. Dashed lines demark the threshold of delta AUC >0.3 or <−0.3.DHeatmap of the screening hits as defined in C. AUC is color coded.E–JDose–response curves of ID188 and ID211 organoids (ID188 p27‐29, ID211 p26‐28) or 2D lines (ID188 p5+29, ID211 p9+13) treated with (E and F): cobimetinib or (H and I): biminetinib. (G) cobimetinib and (J) biminetinib: GI_50_ values. Shown is the mean ± SD of three independent experiments.KIllustration of a cohort of patients (*n* = 15) with available primary 2D PDAC cell lines. This cohort includes the ID188 (pre‐FFX (FOLFIRINOX), red) and ID211 (post‐FFX, blue) lines. These lines were screened for sensitivity toward three MEKi.LFifteen primary human PDAC 2D cell lines were screened for MEKi (as indicated) sensitivity. The determined AUC was variance‐scaled, and the z‐scores are depicted. The ID188 and ID211 identity is color coded. Shown is the median, upper, and lower quartiles as well as upper and lower extremes.

**Figure EV2:**
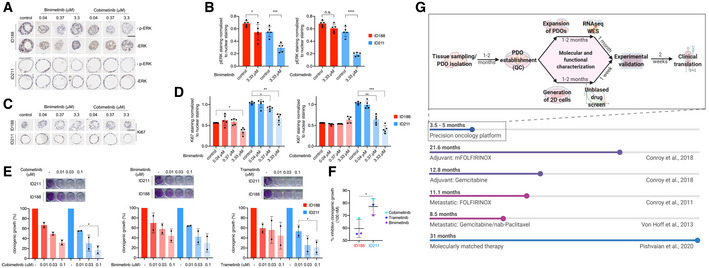
MEKi response pERK IHC staining of embedded and sectioned organoids of ID188 (p30) and ID211 (p30) upon 24 h of binimetinib and cobimetinib treatment. Scale bar = 50 µm.Quantification of the pERK staining normalized to nuclear staining. Shown is the mean ± SD of *n* = 5 organoid images. Paired *t*‐test: n.s. = not significant, *P < 0.05, ***P < 0.001, and ****P < 0.0001.Ki67 IHC staining of embedded and sectioned organoids of ID188 (p30) and ID211 (p30) upon 24 h of binimetinib and cobimetinib treatment. Scale bar = 50 µm.Quantification of the Ki67 staining normalized to nuclear staining. Shown is the mean ± SD of *n* = 5 organoid images. ANOVA test: *P < 0.05, **P < 0.01, and ***P < 0.001.Clonogenic assay of the indicated 2D lines (ID188 p5+29–36, ID211 p9+13–16). Upper panel: illustration of one out of two clonogenic assays. Lower panel: quantification; Shown is the mean ± SD of *n* = 2 independent experiments. ANOVA test: *P < 0.05.Percent inhibition of clonogenic growth at 100 nM trametinib, binimetinib, or cobimetinib was calculated. Unpaired *t*‐test: *P < 0.05. Shown is the mean ± SD of *n* = 3 biological replicates.Timeline from tissue sampling to clinical translation of our longitudinal pipeline in context of the median survival observed with standard of care therapies.

We next asked whether chemotherapy‐induced changes were functionally relevant and determined the drug response in our models. Here, we observed reduced FOLFIRINOX sensitivity of ID211 organoids, arguing that the chemotherapy applied *in vivo* was inducing a resistance phenotype (Fig 2A). In order to identify therapy‐induced vulnerabilities, we performed an unbiased drug screen with a library containing 415 compounds using 2D lines ID188 and ID211 applicable to our automated liquid‐handling device. We treated cells with a seven‐point dilution over 72 hours and used ATP as a surrogate for the drug response. The area under the dose response curve (AUC) was used as a measure for sensitivity (Fig 2B and Dataset EV3). Applying a delta AUC of +/‐0.3 as a threshold to define a relevant change in the sensitivity, we observed that the response of 3% of the investigated drugs was different (Fig 2C). Cells isolated after FOLFIRINOX (ID211) were resistant to Tozasertib (AURORA kinase inhibitor), Birinapant and LCL161 (SMAC mimetics), and 4EGI‐1 (eIF4E/eIF4G interaction inhibitor) (Fig 2D). In contrast, these cells showed increased sensitivity to three groups of drugs, which include inhibitors of the ATPase p97/valosin containing protein (VCP) (CB‐5083, NMS‐873), the epidermal growth factor receptor (EGFR) (lapatinib, poziotinib), and MEK (trametinib, cobimetinib, BI‐847325) (Fig 2D). Since canonical KRAS‐MEK‐ERK signaling is a major driver pathway and relevant target in PDAC (Waters & Der, 2018), we validated the MEK inhibitors (MEKi). We used an additional MEKi, binimetinib, whose delta AUC was 0.27 in the screening experiment. In the 2D and PDO lines (ID211) isolated after the FOLFIRINOX therapy, an increased sensitivity to cobimetinib (Fig 2E–G) and binimetinib (Fig 2H–J) was detected, confirming the screening results. The cobimetinib GI_50_ (= half maximal growth inhibitory concentration) was 10–18‐fold reduced (Fig 2G) in the ID211 models, the binimetinib GI_50_ (Fig 2J) 9–12‐fold. On‐target activity of both MEKi was validated by measuring phosphorylation of ERK as a surrogate (Fig EV2A). Quantification of ERK phosphorylation showed an augmented inhibition induced by both MEKi in sensitive ID211 PDOs (Fig EV2B). Consistently, the Ki67 index was dose‐dependently reduced by both MEKi in sensitive ID211 PDOs, but only affected by high concentrations in resistant ID188 PDOs (Fig EV2C and D). Also, in 2D models, increased sensitivity of ID211‐derived cancer cells toward three MEKi was observed in colony formation assays, confirming data of the viability assays (Fig EV2E and F).

To place these results into a larger context, we analyzed the MEKi response of additional 15 primary patient‐derived lines as illustrated in Fig 2K. For all MEKi, the cells isolated before the FOLFIRINOX therapy (ID188) belong to the MEKi resistant group, whereas the cellular model isolated from the resected PDAC (ID211) is the most MEKi sensitive line (Fig 2L). Therefore, the induced vulnerability is highly relevant in context of a larger cohort of primary PDAC cell lines, validating the initial *N‐of‐1* approach.

Interestingly, SNAI2, an EMT transcription factor, was recently connected to MEKi resistance of PDAC (Bilal *et al*, 2021). Therefore, increased expression of *SNAI2* mRNA observed in ID188 organoids (Fig EV1E) might contribute to the phenotype. However, detailed deciphering of the molecular process explaining the resistance phenotype demands additional experiments.

We show here that despite the primary PDAC models sharing their genetic drivers and both being classified as classical subtype, they fundamentally differ phenotypically and in the responsiveness toward polychemotherapy, as well as relevant targeted therapies. Importantly, the genetic landscape between the investigated models is not altered significantly, preventing selection of a therapy by a genetic driver. Therefore, in the presented study, neither PDAC subtyping nor genomics allowed to apply molecularly informed therapies. In contrast, therapeutic vulnerabilities were identified by unbiased drug screening experiments. We interpreted these results as a need for an additional functional layer for precision oncology. With continuously improving culture and screening techniques, we argue that functional layers should be implemented into precision oncology programs to selected PDAC patients, such as patients in a neo‐adjuvant therapeutic setting.

The clinical case presented demonstrates impressively that the biology of a PDAC is skewed substantially in response to polychemotherapy, which is also reflected by altered drug sensitivities. Clear evidence shows that drug‐induced vulnerabilities can be therapeutically exploited, and clinical testing of such a concept is ongoing (NCT02836548) (Vecchione *et al*, 2016; Wang *et al*, 2018, 2019). We observed a group of drugs, p97/VCP inhibitors, EGFR inhibitors, or MEKi, with higher sensitivity in PDAC models after the FOLFIRINOX treatment. This finding mandates a systematic investigation of secondary sensitivities occurring after FOLFIRINOX to implement targeted maintenance or second‐line therapies.

Previous investigations into treatment‐imposed plasticity in primary patient–derived PDAC cells showed a rather uniform activation of a basal‐like / EMT state occurring upon the treatment with FOLFIRINOX (Porter *et al*, 2019). Here, although both models investigated were classified as classical PDAC, the cellular models isolated after the FOLFIRINOX therapy show signs of a re‐differentiation process, deduced from the different growth pattern and the loss of molecular signatures associated with the basal‐like subtype. Interestingly, a switch from a more aggressive basal‐like subtype to a more differentiated classical subtype under an ongoing chemotherapy has been described (Chan‐Seng‐Yue *et al*, 2020; Grünwald *et al*, 2021). Such data underscore the pronounced plasticity of PDAC. Such plasticity processes may allow the tumor to switch to the subtype resistant to the applied chemotherapy and illustrate the need to understand these molecular events. The events directing plasticity are often mediated by epigenetic regulation and chromatin remodeling, and their understanding is of great value to establish plasticity blocking therapies.

Overall, many processes contribute to the establishment of resistance, including the clonal evolution of the tumor under therapeutic selection pressure; however, non‐genetic mechanisms are increasingly recognized to confer therapy resistance (Marine *et al*, 2020). It will be important to understand the determinants deciding for the usage of genetic versus non‐genetic roads to resistance and to develop predictability models, which will allow to interfere with resistance development.

Importantly, retrospective studies suggest that transcriptomic signatures of PDOs and PDCL have value in predicting response to chemotherapy in PDAC (Tiriac *et al*, 2018; Nicolle *et al*, 2020). Hereby, a combination of PDOs and PDCLs as models to define predictive biomarkers may be superior to the use of only one (Nicolle *et al*, 2021). However, it remains to be shown how these signatures perform in a longitudinal setting after chemotherapy, considering plasticity reprogramming.

As indicated above, real‐world outcomes suggest that genetic profiling followed by molecularly tailored therapy is prolonging overall survival of PDAC patients (Pishvaian *et al*, 2020). At the same time, only 26% of PDAC patients present with actionable alterations (Pishvaian *et al*, 2020). Considering the median survival of current standard of care in adjuvant and palliative regimens (Fig EV2G), our functional workflow, although technically challenging and time consuming, is applicable to PDAC patients regardless of their genetic profile and, additionally, useful after acquisition of therapeutic resistance.

In summary, we introduce a functional longitudinal precision oncology platform which integrates clinical and multimodality imaging data, a detailed genomic and transcriptomic analysis, as well as functional investigation. Importantly, we identify altered chemotherapy‐induced vulnerabilities without a selectable genetic marker. Specifically, we identify sensitivity toward MEKi, driven by tumor plasticity in response to FOLFIRINOX treatment. Our data support the hypothesis that functional data will expand applications of precision oncology.

## Materials and Methods

### Clinical data

The study was conducted in accordance with the Declaration of Helsinki and conformed to the Department of Health and Human Services Belmont Report. Approval by the local ethics committee (Project 207/15, 1946/07, 330/19S, 181/17S, 5542/12 and 80/17S) was given, and written informed consent was obtained from the patient prior to the investigation.

The following clinical data were obtained for the patient using the hospital’s information system: sex, age at diagnosis, tumor markers CEA and CA 19‐9, tumor formula, type of chemotherapy (neoadjuvant, adjuvant) and chemotherapeutic regime (FOLFIRINOX). Clinical evaluation of tumor size, lymph node status, and metastasis (TNM Classification Edition 8, 2017) was performed on baseline CT before starting the treatment and in follow‐up examinations.

### Generation and culture of patient‐derived organoids and 2D cell lines

Primary patient‐derived PDAC 3D organoids were generated from EUS‐guided fine needle biopsy (EUS‐FNB) and surgical resection as it was described previously (Dantes *et al*, 2020). Briefly, biopsies were minced into small pieces, and surgery specimens were incubated rotating for collagen digestion using a digestion buffer (DMEM‐F12 (#31330095, Thermo Fisher Scientific, Massachusetts, USA), 1 × primocin (#ant‐pm‐2, Invivogen, San Diego, USA), and 6 mg/ml collagenase II (#17101015, Thermo Fisher Scientific)). Tissue pellets were incubated for 3–10 min with red blood cell lysis buffer (#A1049201, Thermo Fisher Scientific) and afterward further digested using TrypLE (#12604039, Thermo Fisher Scientific). Last, cell pellets were resuspended in 50 µl of Matrigel/well (#354230, Corning Life Sciences, Corning, USA) and PDO medium (DMEM‐F12 (#11320033, Thermo Fisher), 5mg/ml D‐Glucose (#G8270, Sigma‐Aldrich, St. Louis, USA), 0.5% ITS Premix (#354350, Corning Life Sciences), 5 nM 3,3,5‐triiodo‐L‐thyronine (#T0821, Sigma‐Aldrich), 1 µM dexamethasone (#D175, Sigma‐Aldrich), 100 ng/ml cholera toxin (#C9903, Sigma‐Aldrich), 1% penicillin/streptomycin (#15140122, Thermo Fisher Scientific), 5% NU‐Serum IV (#355500, Corning Life Sciences), 25 µg/ml bovine pituitary extract (#P1167, Sigma‐Aldrich), 10 mM nicotinamide (#N3376, Sigma‐Aldrich), 100 µg/ml primocin (#ant‐pm05, Invivogen), 0.5 µm A83‐01 (#2939, Tocris, Bristol, UK), 10% RSPO1‐conditioned medium (R‐spondin‐1 overexpressing cell line HEK293T, provided by the Hubrecht Institute (Uppsalalaan 8, 3584 CT Utrecht, Netherlands), 100 ng/ml recombinant human heregulin‐1 (#100‐03, Peprotech, Cranbury, USA), and 10 µM rho kinase inhibitor (#TB1254‐GMP, Tocris) was added 10 min later. For passaging, the media were aspirated and 250 µl of cell recovery solution (#11543560, Corning Life Sciences) was added to each well for 5 min. Subsequently, this mixture was dissolved in 1 ml of ice‐cold PBS (#14190144, Thermo Fisher Scientific) supplemented with 0.1% BSA (#11930, Serva Electrophoresis GmbH, Heidelberg, Germany). After 30 min on ice, organoids were centrifuged at 1,000 rpm at 4°C for 5 min and washed and centrifuged again. Thereafter, cell pellets were resuspended in 50 µl Matrigel/well (#354230, Corning Life Sciences), and the medium was added 10 min later.

In order to make PDOs applicable to high‐throughput drug screening using a liquid‐handling robot, we generated 2D lines from PDOs using the outgrowth method. Briefly, after the establishment of 3D organoids from primary PDAC specimens (ID188 at passage 5 and ID211 at passage 9), 2D cells were allowed to grow out from the Matrigel and attach to the plastic. Matrigel and organoids were removed, and 2D cells were further cultured using RPMI medium (#21875091, Thermo Fisher Scientific) supplemented with 10% fetal bovine serum (#10270106, Thermo Fisher Scientific) and 1% penicillin/streptomycin (#15140122, Thermo Fisher Scientific). For passaging, the medium was removed, cells were washed with PBS (#14190144, Thermo Fisher Scientific), and trypsinized using 1–2 ml of 1× trypsin (#15400054, Thermo Fisher Scientific) for 5–10 min. In order to stop trypsinization, new medium was added, and cells were distributed into new flasks or seeded for experiments.

In addition to primary patient‐derived PDAC 2D cells described above, cell lines HuPDAC1, HuPDAC3, HuPDAC7, HuPDAC11, HuPDAC14, and HuPDAC17 were cultured in RPMI GlutaMAX^®^ (#61870036, Life Technologies, Darmstadt, Germany) supplemented with 20% fetal bovine serum (#S 0615, Biochrom GmbH, Berlin, Germany) and 1% penicillin/streptomycin (#15140122, Thermo Fisher Scientific). The patient‐derived lines PDC40, PDC49, PDC56, PDC117, and PDC148 were cultured in Advanced DMEM F‐12 (#12634010, Thermo Fisher Scientific) supplemented with 10% fetal bovine serum (#S 0615, Biochrom GmbH) and 1% penicillin/streptomycin (#15140122, Thermo Fisher Scientific).

### Pharmacotyping

For pharmacotyping of 3D cells, organoids with passages between 26 and 30 were processed as abovementioned and digested to a single‐cell suspension using 1× trypsin. 500 cells/well were seeded in a total volume of 20 µl/well (2 µl Matrigel + 18 µl PDO media) in a 384‐well plate (#CLS3765, Corning Life Sciences). After 24 h, cobimetinib and binimetinib (SelleckChem, Houston, USA) were added in a 7‐point drug dilution to the cells (highest concentration = 10 µM) and incubated for 72 hours. For the *in vitro* FOLFIRINOX treatment, a mixture of 5‐fluoruracil (c_max_ = 37.6 µM), irinotecan (c_max_ = 16.9 µM), and oxaliplatin (c_max_ = 7.9 µM) was prepared according to the ratio in clinical practice and added in a 7‐point drug dilution for 72 h. In order to measure viability, 5 µl of the CellTiter‐Glo^®^ Luminescent Cell Viability reagent (#G7573, Promega, Madison, USA) was added and incubated for 15 min shaking. Luminescence was measured on a FLUOstar OPTIMA microplate reader (BMG Labtech GmbH, Ortenberg, Germany).

For pharmacotyping of 2D cells, cells with passages between 9 + 13 to 5 + 29 were cultured in RPMI GlutaMAX^®^ (#61870036, Life Technologies, Darmstadt, Germany) supplemented with 20% fetal bovine serum (#S 0615, Biochrom GmbH) and 1% penicillin/streptomycin (#15140122, Thermo Fisher Scientific). The cells were digested to a single‐cell suspension using 1 × TrypZean^®^ Solution (#T3449, Sigma‐Aldrich). 1500 or 3000 cells/well were seeded in a total volume of 100 µl/well in a 96‐well plate (# 9157BC, Corning Life Sciences). After 24 h, each drug was added in seven concentrations (3‐fold dilution series, highest concentration 10 µM). After 72 h, 25 µl CellTiter‐Glo^®^ Luminescent Cell Viability reagent was added and incubated for 10 min shaking. Luminescence was measured in a CLARIOstar microplate reader (BMG Labtech GmbH).

Values were normalized to their respective DMSO control and IC_50_ values (non‐linear regression model), and area under the curve (AUC) was calculated using GraphPad Prism 8 (GraphPad Software, San Diego, USA, RRID:SCR_002798).

### Proliferation assay

For investigating the growth rates of human 3D cells, organoids with passages between 26 and 30 were processed as mentioned above and digested to a single‐cell suspension using 1× trypsin. 500 cells/well were seeded in a total volume of 20 µl/well (2 µl Matrigel + 18 µl PDO media) in a 384‐well plate. Cell growth was determined for 5 consecutive days using 5 µL of the CellTiter‐Glo^®^ Luminescent Cell Viability reagent.

### Clonogenic assay

3,000–6,000 cells with passages between 9 + 13 to 5 + 36 per well were seeded in 1 ml medium in 24‐well plates. After 24 h, the medium was exchanged, and drugs were added in six different concentrations (highest concentration 3.3 µM, 3‐fold dilution series). The cells were cultured for 1–2 weeks. Afterward, the medium was removed. Cells were washed once with PBS and stained with 200 µl Crystal Violet (#C6158, Sigma‐Aldrich) solution (2% (v/v) EtOH and 0.2% (w/v) Crystal Violet in H_2_O) for 60 min on a shaker at room temperature. After removal of the Crystal Violet solution, cells were washed for the first time with H_2_O for one hour and for a second time overnight on a shaker at room temperature. The plates were dried for several days and subsequently visualized using a flatbed scanner (Seiko Epson K.K., Suwa, Nagano, Japan). For quantification, the Crystal Violet stain was solubilized using 600 µl per well of 1% (w/v) sodium dodecyl sulfate (SDS) (#2326.2, Carl Roth, Karlsruhe, Germany) solved in H_2_O. The plates were incubated overnight on a shaker at room temperature, and absorbance of the solubilized Crystal violet was measured at 595 nm using a CLARIOstar microplate reader (BMG Labtech GmbH).

### Automated drug screening

1,500–3,000 cells per well (depending on growth rate) between passages 9 + 11 and 5 + 27 were seeded in 96‐well plates (#3971, Corning Life Sciences) using a Multidrop™ Combi Reagent Dispenser (Thermo Fisher Scientific). After overnight incubation at 37°C and in 5% CO_2_ in a Cytomat™ 24C automated incubator (Thermo Fisher Scientific), the cells were treated with a compound library using a CyBio^®^ FeliX pipetting platform (Analytik Jena, Jena, Germany). All compounds were obtained from SelleckChem. Each drug was added in seven concentrations (3‐fold dilution series, highest concentration 10 µM). Cell viability was measured after 72 h using CellTiter‐Glo^®^ Luminescent Cell Viability Assay. The reagent was added using a Multidrop™ Combi Reagent Dispenser (Thermo Fisher Scientific). After incubation for 10 min, luminescence was measured in an Infinite^®^ 200 PRO microplate reader (Tecan Group AG, Männedorf, Switzerland). Drug response was analyzed using the R package GRmetrics (Hafner *et al*, 2016; Clark *et al*, 2017).

### Imaging protocol and analysis

Simultaneous ^18^F‐FDG PET/MRI was performed using an integrated whole‐body 3T PET/MRI system (Siemens Biograph mMR, Siemens Healthcare, Erlangen, Germany). The patient was instructed to fast for at least 6 h before injection of 400 MBq (baseline) and 393 MBq (follow‐up) ^18^F‐FDG injection. In addition, 20 mg furosemide as well as oral contrast (Telebrix, 15 ml on 1l) were applied. The PET/MRI scans were started 51 min (baseline) and 63 min (follow‐up) after tracer injection.

PET/MRI examination of the pancreas was performed simultaneously within a 20‐min PET scan of the upper abdomen. A T1‐VIBE Dixon sequence was used for attenuation correction. Further MRI sequences included an axial and coronal T2 haste sequence, axial fat saturated (FS) T2 haste sequence, axial DWI (b‐values 0, 50, 300, and 600 s/mm^2^), axial T1 VIBE Dixon sequence in breath‐hold before and after dynamic administration of contrast agent (arterial, venous and late venous phase) with gadolinium (0,2 ml/kg bodyweight), and an axial T1 VIBE Dixon FS after contrast administration.

PET data were reconstructed using a vendor‐provided iterative reconstruction algorithm (three iterations, 21 subsets, image matrix 172 × 172, zoom 1, gauss filter, full width at half maximum 4.0 mm, and relative scatter correction). Image analysis was performed by one radiologist with 3 years of experience (FNH) under supervision of a board‐certified expert abdominal radiologist with 10 years of experience (RFB) as well as a board‐certified expert nuclear medicine physician with 10 years of experience (ME).

All images were analyzed using OsiriX (OsiriX DICOM viewer, 11.0 OsiriX Foundation; Geneva, Switzerland). Reviewing the axial T2w, DWI, and ADC together with the PET images, the tumor localization was identified. The maximum as well as the peak standardized uptake values (SUV_max_ and SUV_peak_; in g/ml) were obtained from the tumor.

### Histology

For histological analyses, the FNB sample and surgical resection were embedded into paraffin and further processed for pathological investigation in the pathology department at Klinikum rechts der Isar. For histological analysis of organoids, PDOs with passage 30 were fixed for 15 min with 4% PFA, paraffin‐embedded, and sectioned. H&E staining was performed on deparaffinized sections with Eosin and Mayer’s hematoxylin according to a standard protocol for routine diagnostics.

### Immunohistochemistry

Immunohistochemistry (IHC) staining of paraffin‐embedded and sectioned organoids (passage 30) was performed at the Comparative Experimental Pathology Department at Klinikum rechts der Isar using a Leica Bond RXm system (Leica Biosystems, Nußloch, Germany). Therefore, slides were briefly deparaffinized using a deparaffinization solution, and epitope retrieval was performed using a citrate buffer with pH 6 or ETDA buffer with pH 9 for 20–40 min. The primary antibodies Ki67, GLUT1, pERK, and ERK were diluted and applied for 15 min. Antibody binding was detected using the polymer refine detection kit (#DS9800, Leica Biosystems, RRID:AB_2891238) and visualized after 10 min of incubation with DAB as a dark brown precipitate. Counterstaining was performed using hematoxylin for 5 min. Dehydration was manually performed by alcohol washes with increasing concentrations (70, 96, and 100%) and a final xylene incubation. Afterward, slides were mounted with coverslips using Pertex mounting medium and scanned with 40× magnification. Quantification was performed on five organoid images per condition and cell line using Fiji 2.1.1 (RRID:SCR_002285). Therefore, the plugin for color deconvolution was used to separately quantify the expression of Ki67, pERK, as well as ERK and hematoxylin staining. Finally, the respective protein expression was normalized to hematoxylin in order to obtain relative expression levels.

Ki67 (#ab16667, Abcam, Cambridge, UK, 1:50, RRID:AB_302459).

Phospho‐p44/42 (pErk1/2, #4376, Cell Signaling Technology, Danvers, USA, 1:1000, RRID:AB_331772).

p44/42 MAPK (Erk1/2, #4695, Cell Signaling Technology, 1:100, RRID:AB_390779).

GLUT1 (#ab115730, Abcam, 1:750, RRID:AB_10903230).

### Western blotting

For harvesting protein in 2D cells, cells (ID188 p5+33 and ID211 p9+15) were cultured in a 10‐cm dish until they reached around 80% confluency. After 24 h of treatment with different concentrations of cobimetinib and binimetinib (3.3, 0.3, 0.04 µM), medium was removed, and cells were washed twice with PBS. Depending on the number of cells, 150 to 200 µl of cold RIPA lysis (#ab156034, Abcam) buffer supplemented with protease inhibitor (#11873580001, Roche Diagnostics, Basel, Switzerland) and phosphatase inhibitor (#39050, Serva Electrophoresis GmbH) were added, and protein concentration was adjusted accordingly using 4× Laemmli buffer (#1610747, Bio‐Rad Laboratories Inc., Hercules, USA). For denaturation, the samples were heated at 95°C for 5 min. To allow the separation of proteins, SDS‐PAGE was performed with 12% gels at 100 V for approximately 1.5–2 h in running buffer (25 mM Tris, 192 mM Glycine, 0.1% SDS in H2O). After gel electrophoresis, proteins were transferred onto a nitrocellulose membrane (#10600011, GE Healthcare Life science, Marlborough, USA) using a wet blot system (Bio‐Rad Laboratories Inc.). Blotting was carried out at 350 mA for 1.5 h in transfer buffer (25 mM Tris, 192 mM Glycine, 20% methanol in H2O adjusted to pH 8.3). Afterward, membranes were blocked with 5% skim milk (#T145.3, Carl Roth, Karlsruhe, Germany) in PBS at RT and then incubated with the primary antibodies and diluted in 5% skim milk, overnight at 4°C. After washing membranes three times with PBS‐Tween (#9127.2, Carl Roth) for 5 min, they were incubated with the secondary antibody, diluted in 5% skim milk for 1h at RT in the dark, and again washed three times. Proteins were detected at 700 nm and 800 nm wavelength using an Odyssey^®^ Infrared Imaging System (LI‐COR Biosciences, Lincoln, USA, RRID:SCR_013430). Protein quantification was performed using the Image Studio Lite Software (LI‐COR Biosciences, RRID:SCR_013715), and protein expression values were normalized to the expression of a housekeeping protein.

GATA6 (#5851, Cell Signaling Technology, 1:1000, RRID:AB_10705521).

E‐Cadherin (#3195, Cell Signaling Technology, 1:1000, RRID:AB_2291471).

Keratin 81 (#100929, Santa Cruz Biotechnology, Dallas, USA, 1:500, RRID:AB_2132772).

β‐Actin (#A5316, Sigma‐Aldrich, 1:1000, RRID:AB_476743).

IRDye^®^ 680RD Donkey anti‐Rabbit IgG (#926‐68073, LI‐COR Biosciences, 1:10000, RRID:AB_10954442).

IRDye^®^ 800CW Donkey anti‐Rabbit IgG (#926‐32213, LI‐COR, Biosciences, 1:10000, RRID:AB_621848).

IRDye^®^ 680RD Donkey anti‐Mouse IgG (#926‐68072, LI‐COR, Biosciences, 1:10,000, RRID:AB_10953628).

### Immunofluorescence

For the immunofluorescence (IF) staining, organoids with passage 30 were washed and fixed with 4% PFA for 15 min at RT and treated with 0.15% glycine for 5 min followed by a 2‐min permeabilization using 0.2% Triton‐X 100 in PBS. After washing, cells were blocked in 10% donkey serum and 0.1% BSA diluted in PBS overnight at 4°C. Afterward, phalloidin (#65906, Phalloidin‐Atto 647N, 1:250, Sigma‐Aldrich) was incubated for 2.5 h at RT in the dark. Thereafter, cells were treated for 2 min with DAPI (#D9542, 0.03 µl/ml, Sigma‐Aldrich) and washed and imaged using the Leica TCS SP8 Confocal Microscope (Leica Biosystems).

### Whole‐exome sequencing

Genomic DNA was isolated from both organoid lines (ID188 passage 7, ID211 passage 6) and blood as reference tissue using the AllPrep^®^ DNA/RNA micro kit (#80284, Qiagen, Hilden, Germany), according to the manufacturer’s instructions. DNA concentration was fluorimetrically determined using the Qubit 3.0 system (Thermo Fisher Scientific). Library for whole‐exome sequencing were prepared using the Agilent SureSelect^XT^ Low‐input exome‐seq Human v7 kit following the manufacturer’s instructions. Samples were sequenced on an Illumina NovaSeq 6000 sequencer, resulting in approximately 140 Mio. 100 bp‐long paired‐end reads per sample. The GATK Best Practice suggestions were followed for alignment and mutation calling. After read trimming using Trimmomatic 0.38 (LEADING:25 TRAILING:25 MINLEN:50), BWA‐MEM 0.7.17 was used to align reads to the human reference genome (GRCh38.p7). Picard 2.18.26 and GATK 4.1.0.0 were used for postprocessing (CleanSam, MarkDuplicates, BaseRecalibrator) using default settings. Somatic mutations were called using MuTect2 v4.1.0.0 (default settings). Mutations with at least two reads supporting the alternate allele and a base coverage of at least 10 in the tumor and germline were kept. Single‐nucleotide variants (SNVs) and insertions/deletions (Indels) ≤ 10 base pairs were annotated using SnpEff 4.3t, based on ENSEMBL 92. Copywriter 2.6.1.2 (default settings) was used for the detection of copy number variations.

### RNA‐sequencing

Approximately five confluent organoid wells were used to harvest RNA for RNA‐sequencing (RNAseq). Organoids (ID188 passage 7, ID211 passage 6) were processed as abovementioned, and the cell pellet was resuspended in RLT buffer supplemented with β‐mercaptoethanol (Parekh *et al*, 2016). Briefly, barcoded cDNA of each sample was generated with a Maxima RT polymerase (Thermo Fisher Scientific) using oligo‐dT primer containing barcodes, unique molecular identifiers (UMIs), and an adaptor. Ends of the cDNAs were extended by a template switch oligo (TSO), and full‐length cDNA was amplified with primers binding to the TSO‐site and the adaptor. The NEB UltraII FS kit was used to fragment cDNA. After end repair and A‐tailing a TruSeq (Parekh *et al*, 2016), the P5 and P7 sites were exchanged to allow sequencing of the cDNA in read1 and barcodes and UMIs in read2 to achieve better cluster recognition. The library was sequenced on a NextSeq (Macosko *et al*, 2015). Reference genome (GRCh38) was used for alignment. Transcript and gene definitions were used according to the GENCODE Version M25.

### Analysis of RNA‐Seq gene expression data

Raw count data from RNA sequencing from each of the two technical replicates for each organoid line were collapsed by summing all counts per gene into one final raw count profile per organoid line. Afterward, the variance was stabilized by applying a regularized log transformation to the data as implemented in the DESeq2 R package while accounting for different library sizes (Love *et al*, 2014).

A log2 fold change gene expression signature was generated between post‐ and pre‐treatment organoid lines and was used as the input for gene set enrichment analysis (GSEA), which was carried out using the fgsea R package (preprint: Korotkevich *et al*, 2021). Gene sets were retrieved from the MSigDb v7.3 (Subramanian *et al*, 2005; Liberzon *et al*, 2015). Enrichment results for select pathways were illustrated using custom R code.

### Molecular subtyping of PDO lines

Continuous classification using probabilities of class membership (Rashid *et al*, 2020) were determined for each PDO line using its normalized RNA‐seq profile. The PurIST single‐sample classification scheme was implemented based on the gene pairs and coefficients provided by the authors using custom R code. Importantly, adjustment of gene expression for total gene length was omitted because of the 3'prime end sequencing protocol described above. For the respective PurIST gene pairs, gene expression values were illustrated in a heatmap comparing pre‐ and post‐treatment PDO lines.

### Statistical analyses

For this study, no randomization and replication of patients was performed as it is a *N‐of‐1* approach. Pathologists and radiologists were blinded for respective analyses to minimize effects of subjective bias. The Shapiro–Wilk normality tests were carried out for normal distribution. Standard deviation where appropriate is indicated in figure legends, and data are shown as the mean ± SD. Paired as well as unpaired *t*‐tests and ANOVA were carried out as indicated in figure legends (*P* values are indicated).

## Author contributions

**Katja Peschke:** Conceptualization; Formal analysis; Validation; Investigation; Methodology; Writing—original draft; Writing—review and editing. **Hannah Jakubowsky:** Conceptualization; Formal analysis; Validation; Investigation; Methodology; Writing—original draft; Writing—review and editing. **Arlett Schäfer:** Investigation; Methodology. **Carlo Maurer:** Formal analysis; Investigation; Methodology. **Sebastian Lange:** Formal analysis; Investigation; Methodology. **Felix Orben:** Investigation. **Raquel Bernad:** Investigation. **Felix N Harder:** Investigation. **Matthias Eiber:** Investigation. **Rupert Öllinger:** Formal analysis. **Katja Steiger:** Investigation. **Melissa Schlitter:** Investigation. **Wilko Weichert:** Investigation. **Ulrich Mayr:** Resources. **Veit Phillip:** Resources. **Christoph Schlag:** Resources. **Roland M Schmid:** Resources. **Rickmer F Braren:** Investigation. **Bo Kong:** Resources. **Ihsan Ekin Demir:** Resources. **Helmut Friess:** Resources. **Roland Rad:** Funding acquisition. **Dieter Saur:** Funding acquisition. **Günter Schneider:** Conceptualization; Data curation; Formal analysis; Supervision; Funding acquisition; Validation; Investigation; Visualization; Writing—original draft; Writing—review and editing. **Maximilian Reichert:** Conceptualization; Data curation; Formal analysis; Supervision; Funding acquisition; Validation; Investigation; Visualization; Writing—original draft; Writing—review and editing.

In addition to the CRediT author contributions listed above, the contributions in detail are:

KP, HJ, GS, and MR conceived and designed the study. KP, HJ, AS, FO, and RB conducted experiments and/or provided experimental support. FNH, ME, and RFB performed PET/MRI imaging and analysis. CM, SL, and RÖ performed RNA and whole‐exome sequencing with subsequent bioinformatic analysis. UM, VP, CS, BK, IED, HF, and RMS performed endoscopic ultrasound or surgery and provided biopsies and study support. KS, MS, and WW performed H&E staining and histopathological analysis. Funding was provided by RR, DS, GS, and MR. KP, HJ, GS, and MR wrote the manuscript. The equally contributing senior/last author position was determined alphabetically. All authors reviewed and approved the manuscript.

## For more information

DFG‐funded Collaborative Research Center SFB1321 “Modelling and Targeting Pancreatic Cancer” ‐ https://sfb1321.med.tum.de/en

## Supporting information



Expanded View Figures PDF

Dataset EV1

Dataset EV2

Dataset EV3

Source Data for Figure 1

## Data Availability

Raw data from RNA sequencing was uploaded to the GEO database: GSE193389. Due to the potential re‐identifiability and in order to ensure adequate protection of personal data and privacy [https://doi.org/10.15252/embr.201948316], we are not able to make raw genomic (DNA) sequencing data available.
