# Supplementary material for: Identification of treatment‐induced vulnerabilities in pancreatic cancer patients using functional model systems
Source: EMBO Mol Med. 2022 Feb 4;14(4):e14876. doi: 10.15252/emmm.202114876 (PMC8988213; doi:10.15252/emmm.202114876)
Supplement: Supplementary file 1 — Expanded View Figures PDF [file EMMM-14-e14876-s003.pdf]

Expanded View Figures

Figure EV1. Characterization of pre- and post-FFX (FOLFIRINOX) models.

A Schematic illustration of the biopsy strategy and downstream applications.
B GLUT1 IHC of embedded and sectioned organoids (p30 in both PDO lines). Scale bar = 50 μm.
C Growth curves of ID188 (p27-29) and ID211 (p26–28) in 3D culture measured using CellTiter Glo assay for five consecutive days. Shown is the mean ± SD of three independent replicates. Unpaired *t*-test *****P* < 0.0001.
D Western blots of E-cadherin, GATA6, and KRT81 in ID188 (p5+33) and ID211 (p9+15) 2D lines. Actin = loading control. *n* = 2.
E *Vimentin* and *SNAI2* mRNA expression retrieved from RNA-seq experiments in ID188 and ID211 organoids. Shown is the mean ± SD of two technical replicates.

 © 2022 The Authors

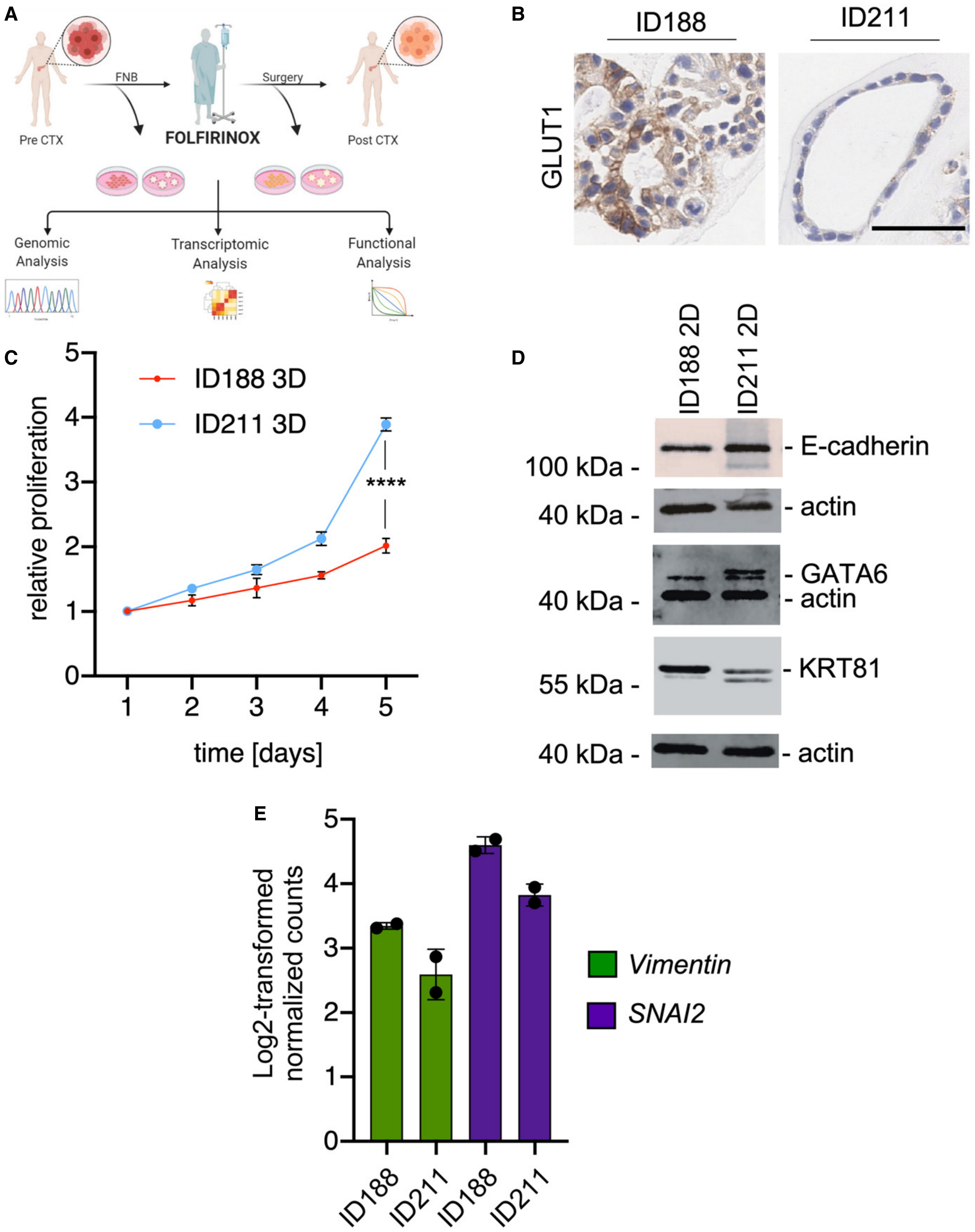
A
FNB
Surgery
Pre CTX
FOLFIRINOX
Post CTX
Genomic Analysis
Transcriptomic Analysis
Functional Analysis
B
ID188
ID211
GLUT1
C
ID188 3D
ID211 3D
relative proliferation
****
time [days]
D
ID188 2D
ID211 2D
100 kDa -
- E-cadherin
40 kDa -
- actin
- GATA6
40 kDa -
- actin
- KRT81
55 kDa -
40 kDa -
- actin
E
Log2-transformed normalized counts
Vimentin
SNAI2
ID188
ID211
ID188
ID211

Figure EV1.

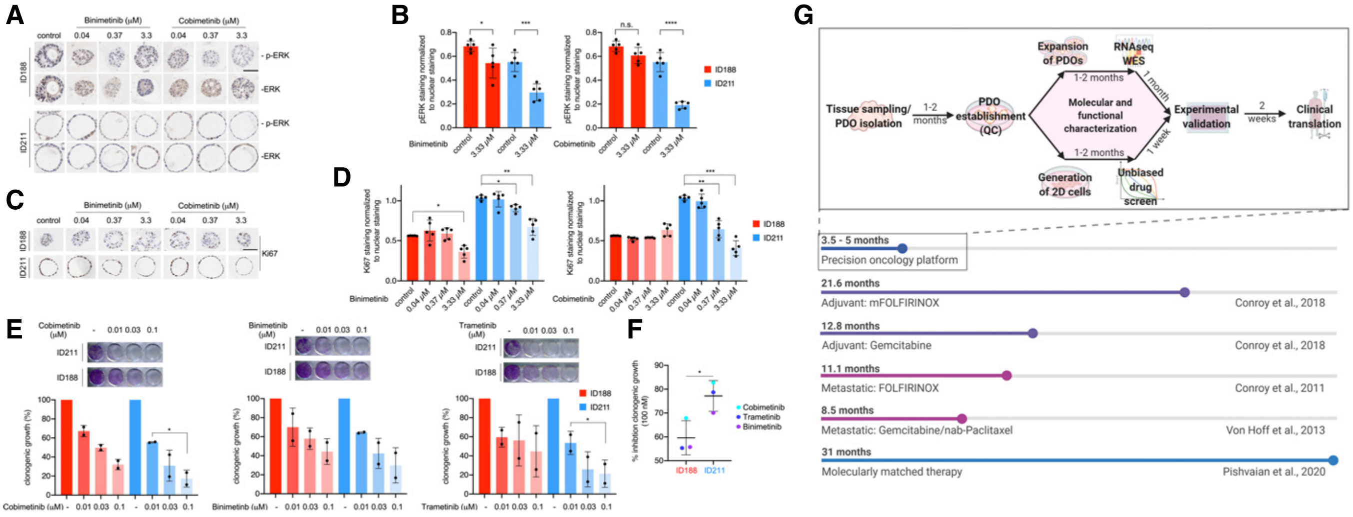

Figure EV2. MEKi response.

A pERK IHC staining of embedded and sectioned organoids of ID188 (p30) and ID211 (p30) upon 24 h of binimetinib and cobimetinib treatment. Scale bar = 50 μm.

B Quantification of the pERK staining normalized to nuclear staining. Shown is the mean ± SD of $n = 5$ organoid images. Paired t-test: n.s. = not significant, $*P < 0.05$, $***P < 0.001$, and $****P < 0.0001$.

C Ki67 IHC staining of embedded and sectioned organoids of ID188 (p30) and ID211 (p30) upon 24 h of binimetinib and cobimetinib treatment. Scale bar = 50 μm.

D Quantification of the Ki67 staining normalized to nuclear staining. Shown is the mean ± SD of $n = 5$ organoid images. ANOVA test: $*P < 0.05$, $**P < 0.01$, and $***P < 0.001$.

E Clonogenic assay of the indicated 2D lines (ID188 p5+29–36, ID211 p9+13–16). Upper panel: illustration of one out of two clonogenic assays. Lower panel: quantification; Shown is the mean ± SD of $n = 2$ independent experiments. ANOVA test: $*P < 0.05$.

F Percent inhibition of clonogenic growth at 100 nM trametinib, binimetinib, or cobimetinib was calculated. Unpaired t-test: $*P < 0.05$. Shown is the mean ± SD of $n = 3$ biological replicates.

G Timeline from tissue sampling to clinical translation of our longitudinal pipeline in context of the median survival observed with standard of care therapies.

 © 2022 The Authors
