# Supplementary material for: Identification of treatment‐induced vulnerabilities in pancreatic cancer patients using functional model systems
Source: EMBO Mol Med. 2022 Feb 4;14(4):e14876. doi: 10.15252/emmm.202114876 (PMC8988213; doi:10.15252/emmm.202114876)

Figure 1 Source data

Uncropped images of HE and IF staining of PDAC tissue and organoids.

Figure 1C

ID188 ID211

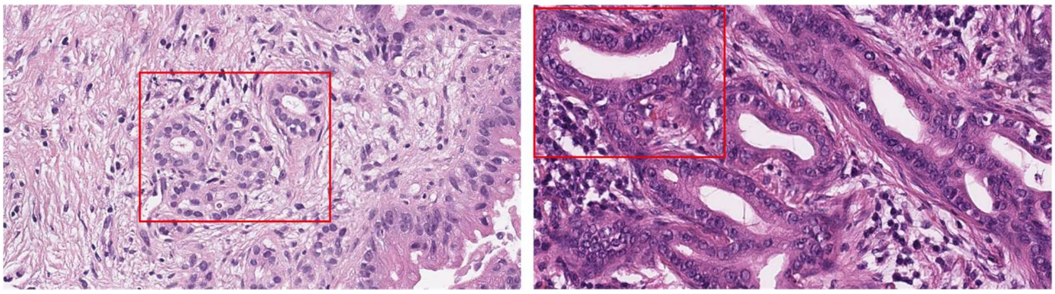

Figure 1E

ID188 ID211

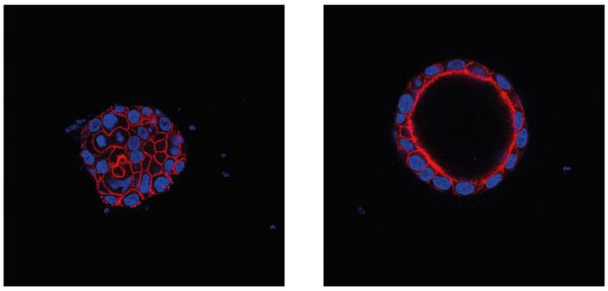

ID188 ID211

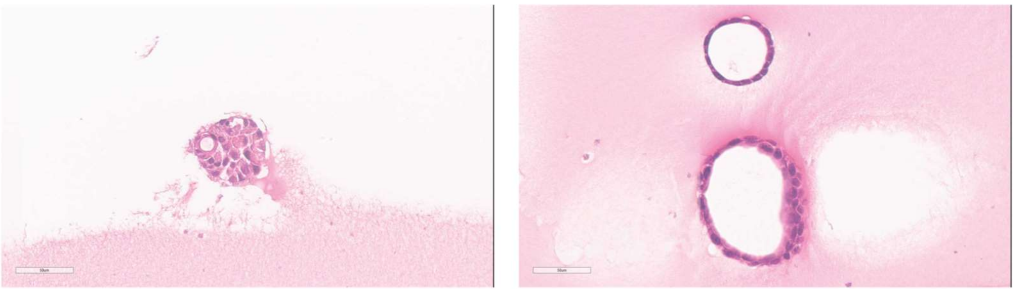

Supplement: Supplementary file 5 — Source Data for Figure 1 [file EMMM-14-e14876-s001.pdf]
