# Supplementary material for: Identification of treatment‐induced vulnerabilities in pancreatic cancer patients using functional model systems
Source: EMBO Mol Med. 2022 Feb 4;14(4):e14876. doi: 10.15252/emmm.202114876 (PMC8988213; doi:10.15252/emmm.202114876)
Supplement: Supplementary file 6 [file EMMM-14-e14876-s006.pdf]

EMBO Molecular Medicine

Identification of therapy-induced vulnerabilities in pancreas cancer using functional model systems

Katja Peschke, Hannah Jakubowsky, Arlett Schäfer, Hans Maurer, Sebastian Lange, Felix Orben, Raquel Bernad, Felix Harder, Matthias Eiber, Rupert Öllinger, Katja Steiger, Anna Schlitter, Wilko Weichert, Ulrich Mayr, Veit Phillip, Christoph Schlag, Roland Schmid, Rickmer Braren, Bo Kong, Ihsan Ekin Demir, Helmut Friess, Roland Rad, Dieter Saur, Günter Schneider, and Maximilian Reichert
DOI: 10.15252/emmm.202114876

Corresponding author: Maximilian Reichert (maximilian.reichert@tum.de)

Review Timeline:

Submission Date:	19th Jul 21
Editorial Decision:	21st Jul 21
Appeal:	21st Jul 21
Editorial Decision:	5th Aug 21
Revision Received:	13th Dec 21
Editorial Decision:	21st Dec 21
Revision Received:	12th Jan 22
Accepted:	13th Jan 22

Editor: Zeljko Durdevic

Transaction Report:

(Note: With the exception of the correction of typographical or spelling errors that could be a source of ambiguity, letters and reports are not edited. Depending on transfer agreements, referee reports obtained elsewhere may or may not be included in this compilation. Referee reports are anonymous unless the Referee chooses to sign their reports.)

1st Editorial Decision 21st Jul 2021

20th Jul 2021

Decision on your manuscript EMM-2021-14876

Dear Prof. Reichert,

Thank you for submitting your manuscript to EMBO Molecular Medicine. Upon receipt, manuscripts can sometimes be evaluated by the Scientific Editors to deal in a timely fashion with a large number of submissions. In this case, I am afraid that we concluded that your manuscript is not well suited for publication in EMBO Molecular Medicine and have therefore decided not to proceed with peer review.

While potentially of interest to the more immediate community, I am afraid that due to its nature, the article doesn't fit well within EMBO Molecular Medicine as we focus primarily on these studies that provide functional novel insights of clinical and/or translational significance, but also that are conceptually novel and of broad interest. As we do not feel that this is the case here, we therefore cannot offer further consideration to your manuscript.

I am sorry to have to disappoint you on this occasion; in the interest of time, I am providing you with an early decision that will allow you to submit your manuscript elsewhere without any further delays.

Please rest assured that this is not a judgment of the quality or interest of your work but a decision based on appropriateness for EMBO Molecular Medicine.

Yours sincerely,

Zeljko Durdevic

Zeljko Durdevic
Editor
EMBO Molecular Medicine

As a service to authors, EMBO provides authors with the possibility to transfer a manuscript that one journal cannot offer to publish to another EMBO publication. The full manuscript and if applicable, reviewers reports are automatically sent to the receiving journal to allow for fast handling and a prompt decision on your manuscript. For more details of this service, and to transfer your manuscript to another EMBO title please click on Link Not Available

Please do not share this URL as it will give anyone who clicks it access to your account.

Dear Dr. Durdevic,

Thank you for considering our manuscript. Certainly, we respect your decision and it is not our common way to rebuttal a decision. However, in this particular case, we both agreed to kindly ask you to reconsider your decision, since it comes as a major suprise to us. We both presented this interdisciplinary work in several talks and traced always an amazing feedback across the fields - even in the zoom age we are living in. As a reviewer EMBO Molecular Medicine for Lise Roth, we are convinced that the manuscript is a very good match for the Journal.

With this work, we are the first group to show how treatment-imposed pressure can be exploited therapeutically in pancreatic cancer and we provide the exact platform for such an approach. For pancreatic cancer, we have clear evidence that precision oncology is a substantial benefit for patients but only 1 out of 4 patients has an actionable genetic lesion. We are the first group clearly demonstrating that functionalizing human organoid models expands the group of patients with a rational therapeutic option. This is an important conceptual advance we are transmitting by our work.

Furthermore, our work gives completely new insights into cellular plasticity in the clinical course of the disease and, therefore, is of high translational significance as well as of broad interest to the field of molecular medicine and personalized oncology. In addition, our work represents a major conceptional advance as we clearly show that tumor de-differentiation is not a default adaptive mechanism in tumor cells being exposed to chemotherapy but rather one of many. Specifically, we demonstrate on a morphologic and molecular level that tumor cells after chemotherapy in vivo are reprogrammed to be more epithelial. Importantly, this reprogramming opens new avenues for targeted therapies. Of note, this reprogramming is not driven by clonal selection and an altered mutational profile. Therefore, we propose to implement patient-derived functional model systems which are able to recapitulate adaptive processes under chemotherapy into the clinical workflow, especially, in highly plastic cancers.

I would be happy to discuss with you on the phone or via zoom, maybe there are certain points I can better explain and put in the right perspective.
When would you be available?

Sincerely,
Max Reichert & Günter Schneider

5th Aug 2021

Dear Prof. Reichert,

Thank you for the submission of your manuscript to EMBO Molecular Medicine. We have now received feedback from the three reviewers who agreed to evaluate your manuscript. As you will see from the reports below, the referees acknowledge the interest of the study but also raise important critique that should be addressed in a major revision.

Further consideration of a revision that addresses reviewers' concerns in full will entail a second round of review. EMBO Molecular Medicine encourages a single round of revision only and therefore, acceptance or rejection of the manuscript will depend on the completeness of your responses included in the next, final version of the manuscript. For this reason, and to save you from any frustrations in the end, I would strongly advise against returning an incomplete revision.

We would welcome the submission of a revised version within three months for further consideration. However, we realize that the current situation is exceptional on the account of the COVID-19/SARS-CoV-2 pandemic. Please let us know if you require longer to complete the revision.

I look forward to seeing a revised form of your manuscript as soon as possible. Use this link to login to the manuscript system and submit your revision: https://embomolmed.msubmit.net/cgi-bin/main.plex

Yours sincerely,

Zeljko Durdevic

Zeljko Durdevic
Editor
EMBO Molecular Medicine

***** Reviewer's comments *****

Referee #1 (Remarks for Author):

The study by Peschke and colleagues describes the development of organoid platform to define tumor response as well as identify second line treatment for pancreatic cancer patient that already went through the first line of treatment (FOLFIRINOX). The manuscript is well written and data elegantly presented. The results included in this version of the manuscript are for the most part supportive of the authors conclusions. However, additional control experiments and larger cohort of organoids are needed to fully establish the clinical usefulness of the organoid platform. See below the specific comments.

1. The authors should demonstrate the targeting efficacy of the MEK inhibitors in the organoid cultures and cell lines experiments. A western blot or IHC/IF analysis of pERK will suffice to address this comment.
2. The FOLFIRINOX in vitro formulation should be clarified. Are 5FU and Irinotecan given simultaneously?
3. The Cell-Titer experiments should be validated by a second method that does not utilize a metabolic marker to measure cell viability.
4. The experiments lack details on the passage of the organoid cultures for each of the results. A copy number profile should be done for each of the passage to make sure that the organoid is genetically stable and remain representative of the primary tumor. It will preferable if the copy number analysis is accompanied by marker analysis using IHC/IF.
5. Most important, these initial findings in two cases should be extended to additional ones to define usefulness of the platform in the clinical setting.

Referee #2 (Comments on Novelty/Model System for Author):

the personalized treatment is necesary for patients with PDAC. This paper describes one realistic possibilty.

Referee #2 (Remarks for Author):

In this paper Katja Peschke and colleagues describes a longitudinal precision oncology platform, which point to the value of mechanistic investigation to advance concepts for PDAC targeting, and report a case to illustrate its efficiency.
This is an original manuscript which describes a promising approach to improve the PDAC treatment particularly by applying drugs which are not still employed in clinics. This type of platform will help in the future to the clinicians to select the better therapeutic strategy for each patient.
I suggest some points to improve this paper before it is accepted.

Comments
This paper demonstrate that changes in sensitivity to drugs were not associated to evident changes in their genetic landscape which, in my opinion, strong suggest that resistance to the treatments is associated to the phenotype of the cells rather they genotype modifications, at least in PDAC. This point merit to be discussed more in details in the manuscript.
Another important point to be discussed is about the place of the molecular markers which should be associated to some chemo-sensitivities ?
Are all the PDACs providers of cells or organoids for applying systematically this approach ? Is the response of each model concordant ? Why select organoids rather primary cell cultures or vice versa ?
In practical terms, what is the necessary time to obtain conclusive results from a give patient? This is determinant for its clinical utilization given the short expectancy of survival of these patients, particularly for non-operable patients.

Referee #3 (Comments on Novelty/Model System for Author):

The strength of the manuscript is the longitudinal analysis of treatment-naive samples and after neoadjuvant therapy in pancreatic ductal adenocarcinoma. Similar approaches have been considered but to the best of my knowledge, there are no longitudinal studies reported. The authors present a pilot study with an integrative analysis of genetics, molecular and pharmacotyping of patient-derived organoids that could be implemented in the management of pancreatic ductal adenocarcinoma.

Referee #3 (Remarks for Author):

The manuscript by Pescheke K, Jakubowsky H, et al., entitled "Identification of treatment-induced vulnerabilities in pancreatic cancer patients using functional model systems" studies the molecular and genetic evolution of tumor cells after neoadjuvant therapy from one patient of pancreatic ductal adenocarcinoma (PDAC). The authors generated tumor-derived organoids and cell lines to investigate genetic and phenotypic adaptations to treatment, and differential drug sensitivity to identify treatment-induced vulnerabilities. They concluded that FOLFIRINOX neoadjuvant treatment induces resistance to FOLFIRINOX and sensitivity to MEK inhibitors independent of a genetic marker. The strength of the manuscript is the longitudinal analysis of tumor samples. Although it is a case study about one patient, which limits the impact in the general population of PDAC patients, the study is timely and relevant as it serves as proof of concept for developing strategies to inform clinical decisions in the management of PDAC.

Major concerns:

The authors claim that therapy induces phenotypic adaptations of tumor cells; however, the data do not exclude pre-existing clones with different sensitivity to FOLFIRINOX in the treatment-naïve sample generated by non-mutational mechanisms. In my opinion, the focus on the phenotypic switch induced by therapy is an oversimplification, and non-mutational mechanisms of tumor heterogeneity should be considered in the interpretation and discussion of the data.

The longitudinal study deserves additional characterization in the diagnostic specimens (pretreatment and resection) and functional model systems. Additional qualitative and quantitative experiments should focus on elucidating epithelial and stromal heterogeneity in naïve and neoadjuvant samples by for example immunofluorescent analysis (e.g., markers of molecular subtypes, fibroblast, and immune infiltration, etc)

I am not convinced that SLUG expression in neoadjuvant treatment explains the acquired sensitivity to MEK inhibitors upon FOLFIRINOX treatment. Additional molecular analysis (e.g., gain and loss of function in patient-derived organoids, etc) should be performed if the authors wish to include this analysis.

Lastly, the authors report a longitudinal precision oncology platform, which could be in principle tested in prospective clinical trials. To strengthen the manuscript's impact and help future studies, the authors could consider including a description of the manpower, technical and funding resources, and time required to implement such a platform in the clinical management of PDAC. Such a description should highlight the limitations as well.

Minor concerns:

• Figure 1 is overcrowded.

• "In an interim staging by 18F-FDG PET-MRI, the glucose metabolism (as measured by the standard uptake value) was markedly reduced while the tumor size was unaltered, indicating a metabolic switch (Fig. 1C)"

Can PET scanning be considered quantitative? Would it be possible that FFX is selectively killing highly cycling cells, which presumably also consume more glucose?

• Provide the nomenclature of the staging system

• "Histologically, both biopsies demonstrated a well to moderate differentiation (Fig. 1D)."

The histological analysis deserves a high resolution image and . Also, is the magnification of ID188 and ID211 the same?

• PDOs isolated pre-chemotherapy (ID188) showed a lumen filling growth pattern and revealed a quasi-mesenchymal growth in 2D (Fig. 1E). PDOs isolated from the resection (ID211) were transparent and grew as an epithelial monolayer with colony forming growth in 2D (Fig. 1E)

Provide a reference were the growth patter of PDOs is associated with molecular subtypes or differentiation.

• PURIST citation is missing "Purity Independent Subtyping of Tumors (PurIST), A Clinically Robust, Single-sample Classifier for Tumor Subtyping in Pancreatic Cancer"

• Fig 1F. Validation of the results by immunofluorescence with markers of classical and basal-like (e.g.; GATA6 and KRT5 respectively)

• Importantly, SNVs with a relevant mutant allele frequency are highly concordant in the investigated models.

The description of SNVs is shallow. What are the implications of MEN1 mutations in the tumor behavior? Highlight the most mutated genes in PDAC

• To place these results into a larger context and to exclude epiphenomena, we analyzed the MEKi response of additional 15 primary patient-derived lines.

What exactly does epiphenomena means in this sentence?

• SLUG. The correct gene symbol is SNAI2

Reviewer 1

1.1. *The authors should demonstrate the targeting efficacy of the MEK inhibitors in the organoid cultures and cell lines experiments. A western blot or IHC/IF analysis of pERK will suffice to address this comment.*

We thank the reviewer for this comment and we totally agree that it is meaningful to confirm the on-target activity of the MEKi in our model system. We have performed immunohistochemistry for ERK and phosphorylated ERK in ID188 and ID211 organoid lines treated with Binimetinib and Cobimetinib. Phosphorylation was reduced by both MEKi in ID188 and ID211. Effects were augmented in the ID211 PDO, furthermore underscoring and contributing to explain the increased sensitivity of the ID211 lines. This Figure has been added to Fig. EV2A/B.

1.2. *The FOLFIRINOX in vitro formulation should be clarified. Are 5FU and Irinotecan given simultaneously?*

The detailed formulation and treatment sequence has been added to the M&M section. We apologize for the brevity previously. Specifically, the following paragraph has been added: For *in vitro* FOLFIRINOX treatment, a mixture of 5-Fluoruracil (c_{max} = 37.6 µM), Irinotecan (c_{max} = 16.9 µM) and Oxaliplatin (c_{max} = 7.9 µM) was prepared according to the ratio in clinical practice and added simultaneously in a 7-point drug dilution for 72 hours.

1.3. *The Cell-Titer experiments should be validated by a second method that does not utilize a metabolic marker to measure cell viability.*

We appreciate this insightful comment. We now have quantified Ki67 in PDOs (see Supplementary Fig. EV2C/D) and have performed clonogenic assays (see Supplementary Fig. EV2E/F) upon MEK inhibition, both assays supporting the increased sensitivity of the ID211 models.

1.4. *The experiments lack details on the passage of the organoid cultures for each of the results. A copy number profile should be done for each of the passage to make sure that the organoid is genetically stable and remain representative of the primary tumor. It will preferable if the copy number analysis is accompanied by marker analysis using IHC/IF.*

The reviewer raises an important issue here. Now, we have indicated the specific passages of primary cells or organoids in each experiment. In addition, we have previously performed whole exome sequencing in early passage PDOs (passages P5-10) and at late passage (above passage 50). We confirmed that the genomic profile of PDOs is fairly stable across passages as exemplified in PDO ID25 below. We still believe that long-term culture and passaging alters organoid biology. Therefore, as QC measure in our translational workflow involving PDOs, we have included in our SOP that all functional assays have to be performed between passage 5 and 30. We have indicated the passage number of each experiment in the revised version of the manuscript.

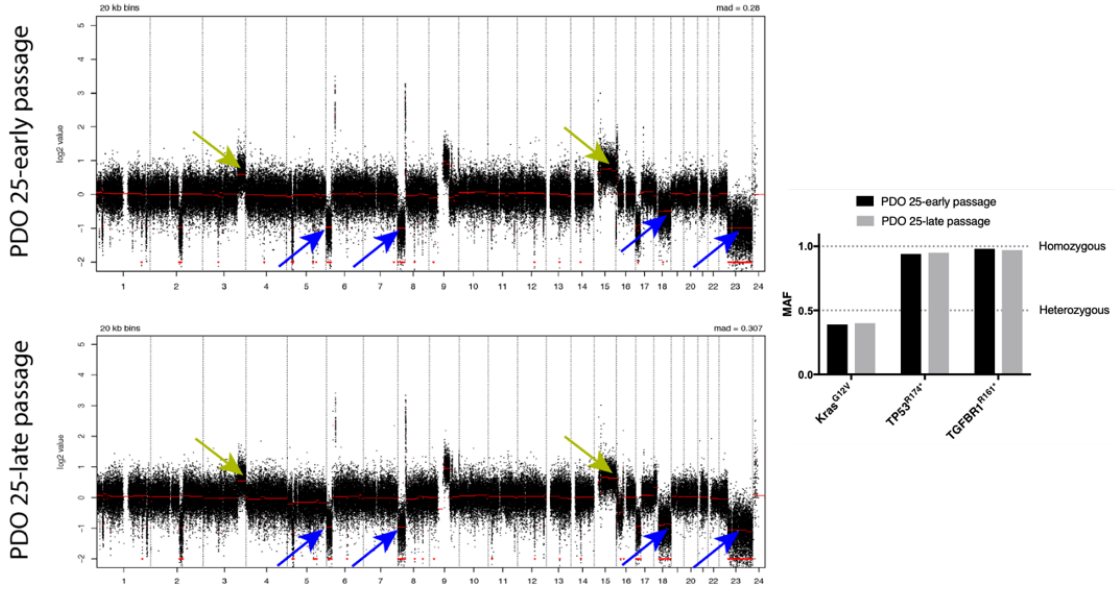

Figure for Reviewer 1: Copy number variations (CNVs) and mutant allele frequency of the indicated oncogenes in early (passage 5) and late passage (>passage 50) PDOs.

1.5. *Most important, these initial findings in two cases should be extended to additional ones to define usefulness of the platform in the clinical setting.*

We agree with the reviewer that our platform and translational implementation of organoid technology in general, needs to pass test of time by validation in co-clinical trials. At the same time, here, we present a *n-of-1 study* to underscore the value of these type of patient-derived model systems facilitating personalized oncology particularly in pancreatic cancer where pure genome-driven approaches frequently fail to identify targeted therapies. To be precise, in the "know your tumor" clinical PDAC trial, in 75% of patient with a complete molecular diagnostic work-up, no targetable lesion was observed. Therefore, we are convinced, with improvements in culture and screening technologies which are currently under development, the functional and mechanistic approach described in the manuscript will fill an important gap. Importantly, the plastic tumor cells behavior in response to chemotherapeutic therapy is not a universal mechanism of resistance or tumor evolution. We know that in certain instances, e.g., clonal selection is a main driver of adaption to treatment. Nevertheless, plasticity is an important mechanism in this context which has been postulated for years and heavily investigated by the basic science community, however, never really shown in a clinical longitudinal setting using patient-derived models. We believe that this is one important strength of our study and we therefore have implemented longitudinal sampling in our standard workflow to expand upon these findings in the near future. In addition, we kindly ask the reviewer to consider data already in the manuscript, which underscore the usefulness of the approach. When we compared the sensitivity of ID188 and ID211 to MEKi to a large panel of patient-derived cell lines, we observed that ID188 is one of the most resistant and ID211 one of the most sensitive lines. To underscore and visualize this finding, we have included an illustration of these important finding as new Fig. 2K.

Reviewer 2

2.1. *This paper demonstrates that changes in sensitivity to drugs were not associated to evident changes in their genetic landscape which, in*

my opinion, strong suggest that resistance to the treatments is associated to the phenotype of the cells rather they genotype modifications, at least in PDAC. This point merit to be discussed more in details in the manuscript.

We thank the reviewer for time, effort and constructive comments. We agree that the description that non-genetic mechanisms contribute to changes in drug sensitivity induced in humans *in vivo*, is a major strength of the work. This will open new research directions, since such data underscore the need to systematically address the molecular underpinning. We increased the discussion and cite a current review article describing the complexity of non-genetic resistance mechanisms.

2.2. *Another important point to be discussed is about the place of the molecular markers which should be associated to some chemo-sensitivities?*

We appreciate the comment and elaborated further on molecular markers and therapy in the discussion section. Specifically, the following sentences have been added: "As indicated above, real-world outcomes suggest that genetic profiling followed by molecularly tailored therapy is prolonging overall survival of PDAC patients (Pishvaian *et al*, 2020). At the same time, only 26% of PDAC patients present with actionable alterations (Pishvaian *et al*, 2020). Our functional workflow, although technically challenging and time consuming, is applicable to PDAC patients regardless their genetic profile and, additionally, useful after acquisition of therapeutic resistance."

2.3. Are all the PDACs providers of cells or organoids for applying systematically this approach? Is the response of each model concordant? Why select organoids rather primary cell cultures or vice versa?

The reviewer makes a key point here. We have included longitudinal sampling to our routine workflow recently. In addition, we have put

additional IRB-approved protocols into place allowing longitudinal sampling not just in a neoadjuvant setting but also at a metastatic stage to explore this important aspect of PDAC biology more systematically in the future.

The main reason for generating 2D lines from PDOs is that our robotic-augmented screening platform allows testing of our 415-drug library of adherent cell in 11 days. Doing this screen in 3D would currently take triple the amount of time and costs. We are working on pipelines to automatize also the 3D screen and reduce the costs for PDO screening. However, so far, we have subsequently validated all hits of the screen in 2D also in 3D organoid culture with extremely high concordance. Having this said, this approach does not exclude false negative results of the high-throughput screen and therefore, robustly validating hits, as exemplified for the MEKi, is necessary. We have added this information in text as well as the supplemental methods.

2.4. In practical terms, what is the necessary time to obtain conclusive results from a give patient? This is determinant for its clinical utilization given the short expectancy of survival of these patients, particularly for non-operable patients.

Again, the reviewer raises the critical issue of life expectancy in these patients. Specifically, in PDAC patients with metastatic disease we have a short window of opportunity in terms of personalized medicine using patient-derived models. Based on our experience screening several PDAC patient-derived cell lines, we have observed that the number of drugs included in the screen could be reduced to approx. 100 individual drugs without significantly reducing the amount of hits. This not just reduced the screening time but more importantly the time of expanding these lines to sufficient cell numbers to perform these types of screens.

In contrast to this, in a neoadjuvant or adjuvant setting due to improved chemotherapy protocols with a median disease-free survival of 21.6 months in the modified-FOLFIRINOX group (Conroy et al., NEJM 2018), there is sufficient time to perform these experiments. At the same time, we have to improve our knowledge on recurrent disease which is also

one reason for emphasizing our longitudinal approach. To clarify the topic raised by the reviewer, we have included the time frame of our longitudinal translational platform in context of the median survival of standard-of-care therapies as new Fig. EV2G.

Reviewer 3

3.1. The authors claim that therapy induces phenotypic adaptations of tumor cells; however, the data do not exclude pre-existing clones with different sensitivity to FOLFIRINOX in the treatment-naïve sample generated by non-mutational mechanisms. In my opinion, the focus on the phenotypic switch induced by therapy is an oversimplification, and non-mutational mechanisms of tumor heterogeneity should be considered in the interpretation and discussion of the data.

We thank the reviewer for the important insights and the efforts to improve our manuscript. We completely agree with the reviewer. We believe non-mutational or epigenetic mechanisms whether they are pre-existing or acquired are so far under-investigated areas of PDAC biology. Our RNAseq results clearly indicate significant changes in transcriptional programs upon treatment. Some of these altered pathways such as TGFbeta signaling have pronounced impact upon morphology and phenotype. We did not intend to oversimplify and reduce the impact of epigenetic mechanisms; however, the phenotype was one of the first findings that jumped to the eye when comparing these PDO lines. In response to the reviewer, we included a paragraph clearly stating that the development of resistance involves multiple processes including evolution under selection pressure of a therapeutic intervention. However, we cite also a recent review article that non-genetic events are currently emerging as contributors of therapy resistance. In sum, we believe that evidence provided by our work will stimulate systematic research into non-genetic events of therapy resistance in PDAC, needed to comprehensively understand all facets of a major clinical problem.

3.2. The longitudinal study deserves additional characterization in the diagnostic specimens (pretreatment and resection) and functional model

systems. Additional qualitative and quantitative experiments should focus on elucidating epithelial and stromal heterogeneity in naïve and neoadjuvant samples by for example immunofluorescent analysis (e.g., markers of molecular subtypes, fibroblast, and immune infiltration, etc)

We thank the reviewer for this comment. Unfortunately, there was no treatment-naïve tissue left as the FNB tissue was used for diagnostics and H&E. We agree that it would have been interesting to study changes in the composition of the tumor microenvironment. Yet again, it is not clear how representative these changes would have been for other patients. To further characterize our patient-derived models we have added additional data on differences in proliferation, GLUT1 expression and expression of EMT markers (see Figure EV1B).

3.3. I am not convinced that SLUG expression in neoadjuvant treatment explains the acquired sensitivity to MEK inhibitors upon FOLFIRINOX treatment. Additional molecular analysis (e.g., gain and loss of function in patient-derived organoids, etc) should be performed if the authors wish to include this analysis.

We agree with the reviewer that the acquired sensitivity towards MEKi is likely caused by multifactorial effects of chemotherapy. SLUG expression has been indicated in this context but as indicated by our gene expression analyses it is way more complex than just EMT reprogramming. Therefore, we decided to move this figure to the supplement and we tune down the statement by including the note that other mechanisms might contribute.

3.4. Lastly, the authors report a longitudinal precision oncology platform, which could be in principle tested in prospective clinical trials. To strengthen the manuscript's impact and help future studies, the authors could consider including a description of the manpower, technical and funding resources, and time required to implement such a platform in the clinical management of PDAC. Such a description should highlight the limitations as well.

Here, the reviewer makes an excellent point regarding feasibility of the platform in a co-clinical trial setting. We have added additional information in the revised manuscript (Fig.EV2G). This illustration places the time frame of our longitudinal translational platform in context of the median survival of standard-of-care therapies. As already stated, we are convinced that for selected patients, such a longitudinal platform will allow mechanistic data (e.g. drug screening) into clinical decision making. In addition, there several strategies to streamline the workflow and increase feasibility as also indicated in response to reviewer 2 (see 2.4).

**Model system**	**Experiment**	**Cell number**	**Time**	**Cost**	**Manpower**
3D	PDO establishment + expansion		2-4 months	500 €	1
3D	RNAseq (duplicates)	1×10^6	1 month		1
3D	WES (sample + blood)	5×10^5	1 month	800 €	1
3D	Unbiased drug screen (100 drugs in duplicates)	8×10^5	1 week	400€	2
3D	Drug validation (1 drug in triplicates)	1.2×10^4	1 week	60 €	1
2D	2D cell establishment + expansion		1-2 months		1
2D	Unbiased drug screen (100 drugs in duplicates)	17×10^6	1 week	500 €	2
2D	Drug validation (1 drug in triplicates)	7.2×10^4	1 week	40 €	1

3.5. Figure 1 is overcrowded.

We thank the reviewer for pointing this out. We are restricted by the Report format to two figures. However, we re-arranged figure 1 to increase clarity.

3.6. "In an interim staging by 18F-FDG PET-MRI, the glucose metabolism (as measured by the standard uptake value) was markedly reduced while the tumor size was unaltered, indicating a metabolic switch (Fig. 1C)" Can PET scanning be considered quantitative? Would it be possible that FFX is selectively killing highly cycling cells, which presumably also consume more glucose?

The reviewer raises an important question regarding quantification of FDG-PET and a potential bias due to selective killing of highly proliferative cells by chemotherapy. For oncologic diagnostic purposes, PET-avid lesions are frequently compared and quantified by measuring the maximum standard glucose uptake value (SUV_{max}). In fact, this imaging was part of a collaborative clinical trial which just recently was published using this parameter as part of a multiparametric analysis to predict treatment response (DOI: 10.1186/s13550-021-00808-4). Therefore, we consider PET scanning as quantitative.
We cannot exclude that FFX is selectively killing highly cycling cells, but we include data in the revised version, demonstrating that the post-CTX 3D model showed increased proliferation (Fig. EV1C, EV2C/D Supplementary Figure 1C and 2C/D). This data argues against a selective killing of cancer cells with high proliferative capacity. Since we cannot exclude differential influence of the culture medium, we do not discuss the possibility.
Taken together, although we cannot revisit and asses the primary tissue for proliferation as none of the FNB tissue is available anymore, our organoids indicate that proliferation is not confounding our conclusions.

3.7. Provide the nomenclature of the staging system

The patient was staged according the TNM Classification Edition 8, 2017.

3.8. "Histologically, both biopsies demonstrated a well to moderate differentiation (Fig. 1D)." The histological analysis deserves a high-resolution image and, also, is the magnification of ID188 and ID211 the same?

We have adjusted the magnification und increased resolution (please see Figure 1).

3.9. PDOs isolated pre-chemotherapy (ID188) showed a lumen filling growth pattern and revealed a quasi-mesenchymal growth in 2D (Fig. 1E). PDOs isolated from the resection (ID211) were transparent and grew as an epithelial monolayer with colony forming growth in 2D (Fig. 1E). Provide a reference were the growth pattern of PDOs is associated with molecular subtypes or differentiation.

We thank the reviewer for this important comment. Indeed, in a recent collaboration, the team demonstrated that oncogenic transformation by *Kras*^{G12D} with or without loss of *CDKN2A* is accompanied by EMT and lumen-filling phenotype in a human pluripotent stem cell-derived organoid model system (Breunig et al., Cell Stem Cell 2021). Similar observations, Hans Clevers made in his 2019 PNAS article (https://doi.org/10.1073/pnas.1911273116) comparing non-tumor and tumor organoids. These references have been added. Regarding subtypes and differentiation please see response 3.11.

3.10. PURIST citation is missing "Purity Independent Subtyping of Tumors (PurIST), A Clinically Robust, Single-sample Classifier for Tumor Subtyping in Pancreatic Cancer"

We apologize for this negligence and provide the appropriate citation in the revised version of the manuscript.

3.11. Fig 1F. Validation of the results by immunofluorescence with markers of classical and basal-like (e.g.; GATA6 and KRT5 respectively)

In concordance with the switch from a lumen-filling phenotype to a spheric, cystic organoid growth pattern, we observed a mesenchymal-to-epithelial-transition (MET) protein expression pattern by western blot indicated by decreased KRT81, increased E-cadherin and GATA6

abundance in the ID211 2D culture (Fig. EV1D). This is concordant with the changes in the mRNA expression of SNAI2 and Vimentin in the 3D models (see Fig. EV1E). These results underscore our overall conclusion of a re-differentiation occurring upon FFX *in vivo*.

3.12. Importantly, SNVs with a relevant mutant allele frequency are highly concordant in the investigated models. The description of SNVs is shallow. What are the implications of MEN1 mutations in the tumor behavior? Highlight the most mutated genes in PDAC

As the reviewer indicated, *MEN1* and *MAP2K4* mutations in PDAC are rare alterations and there is little literature available. We did not intend to provide a sallow description of SNVs. Therefore, we provided an extensive list in the supplement. For visualization purposes, we have indicated the driver mutation $KRAS^{G12D}$ and other possibly important alterations. However, due to the comment of the reviewer we include that statement that *MEN1* and *MAP2K4* are both rare mutations and that this tumor does not harbor other usual suspects such as *TP53* or *SMAD4*.

3.13. To place these results into a larger context and to exclude epiphenomena, we analyzed the MEKi response of additional 15 primary patient-derived lines. What exactly does epiphenomena means in this sentence?

Epiphenomenon in this context refers to the limitation just using PDOs derived from one single patient. How can we be sure that the chemotherapy-induced vulnerability towards MEKi is meaningful or just a secondary, unrelated phenomenon (epiphenomenon) or coincidence? To avoid misunderstandings, we have rephrased the sentence and added an illustration for clarification (Fig. EV2K).

3.14. SLUG. The correct gene symbol is SNAI2

We thank the reviewer for pointing out this mistake which has been corrected in the revised version of our manuscript.

21st Dec 2021

Dear Prof. Reichert,

Thank you for the submission of your revised manuscript to EMBO Molecular Medicine. I am pleased to inform you that we will be able to accept your manuscript pending the following final amendments:

1) Please address all the points raised by the referee #1.
2) In the main manuscript file, please do the following:
- Move supplementary methods to main manuscript file.
- In M&M, add statistical paragraph that should reflect all information that you have filled in the Authors Checklist, especially regarding randomization, blinding, replication.
- In M&M, include a statement that in addition to the principles set out in the WMA Declaration of Helsinki and the experiments also conformed the Department of Health and Human Services Belmont Report.
- Raw data from large-scale datasets (RNA sequencing and WES) should be deposited in one of the relevant databases and made freely available prior the publication of the manuscript. Use the following format to report the accession number of your data:

The datasets produced in this study are available in the following databases:
[data type]: [full name of the resource] [accession number/identifier] ([doi or URL or identifiers.org/DATABASE:ACCESSION])

Please check "Author Guidelines" for more information.
https://www.embopress.org/page/journal/17574684/authorguide#availabilityofpublishedmaterial
3) The Paper Explained: Please provide "The Paper Explained" and add it to the main manuscript text. Please check "Author Guidelines" for more information. https://www.embopress.org/page/journal/17574684/authorguide#researcharticleguide
4) Synopsis: Every published paper now includes a 'Synopsis' to further enhance discoverability. Synopses are displayed on the journal webpage and are freely accessible to all readers. They include separate synopsis image and synopsis text.
- Synopsis text: Please provide a short stand first (maximum of 300 characters, including space) as well as 2-5 one sentence bullet points that summarise the paper as a .doc file. Please write the bullet points to summarise the key NEW findings. They should be designed to be complementary to the abstract - i.e. not repeat the same text. We encourage inclusion of key acronyms and quantitative information (maximum of 30 words / bullet point). Please use the passive voice.
- Synopsis image: Please provide a striking image or visual abstract as a high-resolution jpeg file 550 px-wide x (250-400)-px high to illustrate your article.
- Please check your synopsis text and image and submit their final versions with your revised manuscript. Please be aware that in the proof stage minor corrections only are allowed (e.g., typos).
5) For more information: There is space at the end of each article to list relevant web links for further consultation by our readers. Could you identify some relevant ones and provide such information as well? Some examples are patient associations, relevant databases, OMIM/proteins/genes links, author's websites, etc...
6) Source data: We encourage you to include the source data for figure panels that show essential data. Numerical data should be provided as individual .xls or .csv files (including a tab describing the data). For blots or microscopy, uncropped images should be submitted (using a zip archive if multiple images need to be supplied for one panel). Please check "Author Guidelines" for more information. https://www.embopress.org/page/journal/17574684/authorguide#sourcedata
7) As part of the EMBO Publications transparent editorial process initiative (see our Editorial at http://embomolmed.embopress.org/content/2/9/329), EMBO Molecular Medicine will publish online a Review Process File (RPF) to accompany accepted manuscripts. This file will be published in conjunction with your paper and will include the anonymous referee reports, your point-by-point response and all pertinent correspondence relating to the manuscript. Let us know whether you agree with the publication of the RPF and as here, if you want to remove or not any figures from it prior to publication. Please note that the Authors checklist will be published at the end of the RPF.
8) Please provide a point-by-point letter INCLUDING my comments as well as the reviewer's reports and your detailed responses (as Word file).

I look forward to reading a new revised version of your manuscript as soon as possible.

Yours sincerely,

Zeljko Durdevic

Zeljko Durdevic
Editor
EMBO Molecular Medicine

***** Reviewer's comments *****

Referee #1 (Remarks for Author):

The authors have been responsive to reviewers critiques. The comments have been adequately addressed. However, few minor points that should be modified in the text. First, comments on the cell plasticity and value to determine mechanism of resistance should be included in the text. Also, what is the value of organoids as tool to detect some of the gene signatures shown to predict chemo response. There should be a short comments on this in the discussion.

Referee #3 (Comments on Novelty/Model System for Author):

The manuscript will be relevant for the audience of EMBO molecular medicine

Referee #3 (Remarks for Author):

The authors have addressed all my concerns

We thank the reviewer for the helpful comments. We added the sentence "The events directing plasticity are often mediated by epigenetic regulation and chromatin remodeling and their understanding is of great value to establish plasticity blocking therapies.", to underscore the value of plasticity in adaption and resistance.

Also, what is the value of organoids as tool to detect some of the gene signatures shown to predict chemo response. There should be a short comments on this in the discussion.

As for the gene signatures to predict chemotherapeutic response, we have included the following sentence in the discussion section: "Importantly, retrospective studies suggest that transcriptomic signatures of PDOs and PDCL have value in predicting response to chemotherapy in PDAC (Tiriac *et al*, 2018; Nicolle *et al*, 2020). Hereby, a combination of PDO´s and PDCL as models to define predictive biomarkers may be superior to the use of only one (Nicolle *et al*, 2021). However, it remains to be shown how these signatures perform in a longitudinal setting after chemotherapy considering plasticity reprogramming."

We are pleased to inform you that your manuscript is accepted for publication and is now being sent to our publisher to be included in the next available issue of EMBO Molecular Medicine.

EMBO PRESS

YOU MUST COMPLETE ALL CELLS WITH A PINK BACKGROUND

PLEASE NOTE THAT THIS CHECKLIST WILL BE PUBLISHED ALONGSIDE YOUR PAPER

Corresponding Author Name: Günter Schneider and Maximilian Reichert
Journal Submitted to: EMBO Molecular Medicine
Manuscript Number: EMM-2021-14876-V3

Reporting Checklist For Life Sciences Articles (Rev. June 2017)

This checklist is used to ensure good reporting standards and to improve the reproducibility of published results. These guidelines are consistent with the Principles and Guidelines for Reporting Preclinical Research issued by the NIH in 2014. Please follow the journal's authorship guidelines in preparing your manuscript.

A- Figures
1. Data
The data shown in figures should satisfy the following conditions:

- the data were obtained and processed according to the field's best practice and are presented to reflect the results of the experiments in an accurate and unbiased manner.
- figure panels include only data points, measurements or observations that can be compared to each other in a scientifically meaningful way.
- graphs include clearly labeled error bars for independent experiments and sample sizes. Unless justified, error bars should not be shown for technical replicates.
- if n< 5, the individual data points from each experiment should be plotted and any statistical test employed should be justified
- Source Data should be included to report the data underlying graphs. Please follow the guidelines set out in the author ship guidelines on Data Presentation.

2. Captions

Each figure caption should contain the following information, for each panel where they are relevant:

- a specification of the experimental system investigated (eg cell line, species name).
- the assay(s) and method(s) used to carry out the reported observations and measurements
- an explicit mention of the biological and chemical entity(ies) that are being measured.
- an explicit mention of the biological and chemical entity(ies) that are altered/varied/perturbed in a controlled manner.

- the exact sample size (n) for each experimental group/condition, given as a number, not a range;
- a description of the sample collection allowing the reader to understand whether the samples represent technical or biological replicates (including how many animals, litters, cultures, etc.).
- a statement of how many times the experiment shown was independently replicated in the laboratory.
- definitions of statistical methods and measures:
  - common tests, such as t-test (please specify whether paired vs. unpaired), simple $\chi 2$ tests, Wilcoxon and Mann-Whitney tests, can be unambiguously identified by name only, but more complex techniques should be described in the methods section;
  - are tests one-sided or two-sided?
  - are there adjustments for multiple comparisons?
  - exact statistical test results, e.g., P values = x but not P values < x;
  - definition of 'center values' as median or average;
  - definition of error bars as s.d. or s.e.m.

Any descriptions too long for the figure legend should be included in the methods section and/or with the source data.

In the pink boxes below, please ensure that the answers to the following questions are reported in the manuscript itself. Every question should be answered. If the question is not relevant to your research, please write NA (non applicable). We encourage you to include a specific subsection in the methods section for statistics, reagents, animal models and human subjects.

USEFUL LINKS FOR COMPLETING THIS FORM

http://www.antibodypedia.com
http://1degreebio.org
http://www.equator-network.org/reporting-guidelines/improving-bioscience-research-repor
http://grants.nih.gov/grants/olaw/olaw.htm
http://www.mrc.ac.uk/Ourresearch/Ethicsresearchguidance/Useofanimals/index.htm
http://ClinicalTrials.gov
http://www.consort-statement.org
http://www.consort-statement.org/checklists/view/32-consort/66-title
http://www.equator-network.org/reporting-guidelines/reporting-recommendations-for-tumo
http://datadryad.org
http://figshare.com
http://www.ncbi.nlm.nih.gov/gap
http://www.ebi.ac.uk/ega
http://biomodels.net/
http://biomodels.net/miriam/
http://jjj.biochem.sun.ac.za
https://osp.od.nih.gov/biosafety-biosecurity-and-emerging-biotechnology/
http://www.selectagents.gov/

B- Statistics and general methods

Please fill out these boxes (Do not worry if you cannot see all your text once you press return)

1.a. How was the sample size chosen to ensure adequate power to detect a pre-specified effect size?	N/A
1.b. For animal studies, include a statement about sample size estimate even if no statistical methods were used.	N/A
2. Describe inclusion/exclusion criteria if samples or animals were excluded from the analysis. Were the criteria pre-established?	N/A
3. Were any steps taken to minimize the effects of subjective bias when allocating animals/samples to treatment (e.g. randomization procedure)? If yes, please describe.	N/A
For animal studies, include a statement about randomization even if no randomization was used.	N/A
4.a. Were any steps taken to minimize the effects of subjective bias during group allocation or/and when assessing results (e.g. blinding of the investigator)? If yes please describe.	Blinding of pathologists and radiologists.
4.b. For animal studies, include a statement about blinding even if no blinding was done	N/A
5. For every figure, are statistical tests justified as appropriate?	Yes
Do the data meet the assumptions of the tests (e.g., normal distribution)? Describe any methods used to assess it.	Yes, this was done for seuqnecing data as part of the QC measures.
Is there an estimate of variation within each group of data?	Yes, as indicated in the graphs.

Is the variance similar between the groups that are being statistically compared?	

C- Reagents

6. To show that antibodies were profiled for use in the system under study (assay and species), provide a citation, catalog number and/or clone number, supplementary information or reference to an antibody validation profile. e.g., Antibodypedia (see link list at top right), 1DegreeBio (see link list at top right).	Yes
7. Identify the source of cell lines and report if they were recently authenticated (e.g., by STR profiling) and tested for mycoplasma contamination.	Yes

* for all hyperlinks, please see the table at the top right of the document

D- Animal Models

8. Report species, strain, gender, age of animals and genetic modification status where applicable. Please detail housing and husbandry conditions and the source of animals.	N/A
9. For experiments involving live vertebrates, include a statement of compliance with ethical regulations and identify the committee(s) approving the experiments.	N/A
10. We recommend consulting the ARRIVE guidelines (see link list at top right) (PLoS Biol. 8(6), e1000412, 2010) to ensure that other relevant aspects of animal studies are adequately reported. See author guidelines, under 'Reporting Guidelines'. See also: NIH (see link list at top right) and MRC (see link list at top right) recommendations. Please confirm compliance.	N/A

E- Human Subjects

11. Identify the committee(s) approving the study protocol.	Approval by the TUM ethics committee : Project 207/15, 1946/07, 330/19, 181/17S and 80/17S
12. Include a statement confirming that informed consent was obtained from all subjects and that the experiments conformed to the principles set out in the WMA Declaration of Helsinki and the Department of Health and Human Services Belmont Report.	Yes.
13. For publication of patient photos, include a statement confirming that consent to publish was obtained.	N/A
14. Report any restrictions on the availability (and/or on the use) of human data or samples.	N/A
15. Report the clinical trial registration number (at ClinicalTrials.gov or equivalent), where applicable.	N/A
16. For phase II and III randomized controlled trials, please refer to the CONSORT flow diagram (see link list at top right) and submit the CONSORT checklist (see link list at top right) with your submission. See author guidelines, under 'Reporting Guidelines'. Please confirm you have submitted this list.	N/A
17. For tumor marker prognostic studies, we recommend that you follow the REMARK reporting guidelines (see link list at top right). See author guidelines, under 'Reporting Guidelines'. Please confirm you have followed these guidelines.	N/A

F- Data Accessibility

18: Provide a "Data Availability" section at the end of the Materials & Methods, listing the accession codes for data generated in this study and deposited in a public database (e.g. RNA-Seq data: Gene Expression Omnibus GSE39462, Proteomics data: PRIDE PXD000208 etc.) Please refer to our author guidelines for 'Data Deposition'.<br><br>Data deposition in a public repository is mandatory for:<br>a. Protein, DNA and RNA sequences<br>b. Macromolecular structures<br>c. Crystallographic data for small molecules<br>d. Functional genomics data<br>e. Proteomics and molecular interactions	All data are available from the corresponding author upon reasonable request.
19. Deposition is strongly recommended for any datasets that are central and integral to the study; please consider the journal's data policy. If no structured public repository exists for a given data type, we encourage the provision of datasets in the manuscript as a Supplementary Document (see author guidelines under 'Expanded View' or in unstructured repositories such as Dryad (see link list at top right) or Figshare (see link list at top right).	All data are available from the corresponding author upon reasonable request.
20. Access to human clinical and genomic datasets should be provided with as few restrictions as possible while respecting ethical obligations to the patients and relevant medical and legal issues. If practically possible and compatible with the individual consent agreement used in the study, such data should be deposited in one of the major public access-controlled repositories such as dbGAP (see link list at top right) or EGA (see link list at top right).	All data are available from the corresponding author upon reasonable request.
21. Computational models that are central and integral to a study should be shared without restrictions and provided in a machine-readable form. The relevant accession numbers or links should be provided. When possible, standardized format (SBML, CellML) should be used instead of scripts (e.g. MATLAB). Authors are strongly encouraged to follow the MIRIAM guidelines (see link list at top right) and deposit their model in a public database such as Biomodels (see link list at top right) or JWS Online (see link list at top right). If computer source code is provided with the paper, it should be deposited in a public repository or included in supplementary information.	N/A

G- Dual use research of concern

22. Could your study fall under dual use research restrictions? Please check biosecurity documents (see link list at top right) and list of select agents and toxins (APHIS/CDC) (see link list at top right). According to our biosecurity guidelines, provide a statement only if it could.	N/A
